# Valorization of waste forest biomass toward the production of cello-oligosaccharides with potential prebiotic activity by utilizing customized enzyme cocktails

**DOI:** 10.1186/s13068-019-1628-z

**Published:** 2019-12-10

**Authors:** Anthi Karnaouri, Leonidas Matsakas, Eleni Krikigianni, Ulrika Rova, Paul Christakopoulos

**Affiliations:** Biochemical Process Engineering, Chemical Engineering, Department of Civil, Environmental and Natural Resources Engineering, Luleå University of Technology, Luleå, Sweden

**Keywords:** Non-digestible oligosaccharides, Cellobiose, Processive endoglucanases, Prebiotics, Enzyme hydrolysis, Nanofiltration

## Abstract

**Background:**

Production of value-added materials from lignocellulosic biomass residues is an emerging sector that has attracted much attention as it offers numerous benefits from an environmental and economical point of view. Non-digestible oligosaccharides represent a group of carbohydrates that are resistant to gastrointestinal digestion, and therefore, they are considered as potential prebiotic candidates. Such oligosaccharides can derive from the biomass cellulose fraction through a controlled enzymatic hydrolysis that eliminates the yield of monomers.

**Results:**

In the present study, hydrolysis of organosolv-pretreated forest residues (birch and spruce) was tested in the presence of four cellulases (EG5, CBH7, CBH6, EG7) and one accessory enzyme (LPMO). The optimal enzyme combinations were comprised of 20% EG5, 43% CBH7, 22% *Tt*LPMO, 10% *Pa*Cbh6a and 5% EG7 in the case of birch and 35% EG5, 45% CBH7, 10% *Tt*LPMO, 10% *Pa*Cbh6a and 5% EG7 in the case of spruce, leading to 22.3% and 19.1 wt% cellulose conversion into cellobiose, respectively. Enzymatic hydrolysis was applied on scale-up reactions, and the produced oligosaccharides (consisted of > 90% cellobiose) were recovered and separated from glucose through nanofiltration at optimized temperature (50 °C) and pressure (10 bar) conditions, yielding a final product with cellobiose-to-glucose ratio of 21.1 (birch) and 20.2 (spruce). Cellobiose-rich hydrolysates were tested as fermentative substrates for different lactic acid bacteria. It was shown that they can efficiently stimulate the growth of two *Lactobacilli* strains.

**Conclusions:**

Controlled enzymatic hydrolysis with processive cellulases, combined with product recovery and purification, as well as enzyme recycling can potentially support the sustainable production of food-grade oligosaccharides from forest biomass.

## Background

Prebiotics were first defined as “non-digestible food ingredients that beneficially affect the host by selectively stimulating the growth and/or activity of one or a limited number of bacteria in the colon that can improve the host health” [1]. A potential prebiotic compound can be any food ingredient and it satisfies certain characteristics such as: (1) resistance to gastric acidity, hydrolysis by mammalian enzymes and gastrointestinal absorption, (2) fermentation by intestinal microbiota, (3) selective stimulation of the growth and/or activity of the intestinal bacteria that contribute to health and well-being. According to the first characteristic, resistance to digestion, the prebiotic does not need to be completely indigestible, but significant amounts should be available in the intestine (especially the large bowel) in order to provide a fermentation substrate [2]. Various health benefits are associated with the NDOs intake, such as improved blood lipid metabolism, regulation of gastrointestinal function, prevention and treatment of constipation, increased vitamin synthesis and improved human immunity. Additionally, they can be used as protective agent when bacteria encounter changes of temperature, pH and other growth conditions [3]. Prebiotic intake is possible to be obtained through the diet through certain fruits and vegetables; however, the levels of the natural prebiotics are too low, indicating the need for enhancing the levels of prebiotic intake [4].

A wide variety of dietary carbohydrates are considered as potential substrates for bacterial fermentation, with resistant starch being the most quantitatively important. Additionally, non-starch oligosaccharides, including plant-derived substrates such as pectin, cellulose and hemicellulose, have a large contribution to the group of non-digestible oligosaccharides (NDOs). NDOs are defined as those carbohydrates with a low degree of polymerization (DP) (they contain between 3 and 10 sugar units) and therefore a low molecular weight [5]. Commercial NDOs are mixtures of oligosaccharides with variable DP [6]. The concept of the NDOs first originated from the observation that the anomeric C atom in position 1 or 2 of the monosaccharide units of some dietary oligosaccharides has a configuration that makes their glycosidic bonds non-digestible to the hydrolytic activity of the human digestive enzymes [7]. The main categories of NDOs include carbohydrates in which the monosaccharide unit is fructose, galactose, mannose, arabinose, glucose and/or xylose, as well as galacturonic acid in the case of pectin [7].

Cello-oligosaccharides (COS) are linear oligosaccharides that are composed of β-1,4-linked glucopyranose units. COS comprise a group of novel important functional oligosaccharides with significant interest [8] and many potential applications in the feed and food industry as potential prebiotic compounds [9–11]. These oligosaccharides, as well as the disaccharide cellobiose, have been shown to enhance the growth of lactic acid bacteria, and their use as prebiotics has been suggested [12, 13]. Additionally, they can derive from the most abundant carbon source on Earth: lignocellulose; therefore, they are, potentially, the most abundant available NDOs. Production of food-grade prebiotic COS from lignocellulosic biomass, more specifically from forest farming residues and forest industry by-products, constitutes a novel attractive process that enforces the sustainable use of biomass resources. These residues are currently being burned for energy recovery in power plants and households with a sale price of approx. 50–100 € per ton dry weight and could potentially be utilized for products of higher value. In Sweden, the forest industry has so far been the focus for the development of a bioeconomy. From both a forestry sector and a society point of view, there is a rapidly growing interest in new technologies that can convert renewable, low-cost biomass from the forest into high-value bulk products, e.g., transportation fuels, fine chemicals and materials.

Two main strategies can be used for the production of COS; acid-based and controlled enzyme-based hydrolysis of the cellulose. The latter is considered as more attractive due to the use of milder reaction conditions and lower production of monomers [5]. However, there is still limited information regarding the large-scale production of COS. The cellobiohydrolase belonging to the glycosyl hydrolase family 7 (CBH7) and the endoglucanase of the family 5 (EG5) are two enzymes of pivotal importance for the production of cellobiose from forest materials, such as spruce and birch [13]. Both belong to a group of enzymes called processive, whose main feature of action is that they release soluble products from the chain ends of a cellulose molecule, and moreover, they perform several hydrolysis cycles after the first cleavage by moving on the same cellulose chain [14, 15]. In the present study, we evaluated different processive endoglucanases of bacterial and fungal origin, belonging to GH9, GH6 and GH48 families for their ability to release COS from model substrates and lignocellulosic biomass residues. The most promising candidates were selected and integrated in enzyme cocktails together with CBH7 and EG5, and their synergistic mode of action was examined. Lytic polysaccharide monooxygenase of auxiliary family 9 was also included in the mixture, as this enzyme has been identified as a main component of cellulolytic cocktails due to its ability to facilitate the activity of hydrolases by creating new chain ends, promoting the defibrillation of cellulose fibers and improving the overall degradation process [16, 17]. The biomass-derived COS, consisted mostly of cellobiose, were tested for their ability to stimulate the growth of gut microbiota (*Lactobacilli* and *Bifidobacteria* species). To our knowledge, this is the first study that not only includes the sustainable production of food-grade COS from waste forest residues, but also the confirmation that they can support the growth of different probiotic strains.

## Results

### Screening of processive cellulases and mode of action

#### i. Hydrolysis on cellulosic polysaccharides and lignocellulosic substrates

Different cellulases of bacterial and fungal origin were screened for their mode of action, and the product profile on both polysaccharidic substrates and oligosaccharides was determined. The enzymes were selected from the enzyme portfolio of NZYTech Lda. (Portugal), based on their potential processive activity. Ten enzymes are classified in the glycoside hydrolase family 9 (GH9), two in GH48, one from GH6, and one from GH5 family. The percentage of cellobiose (*C*2) out of the total products released from the hydrolysis of three polysaccharides with different cellulose crystallinity, namely CMC, PASC and Avicel, is summarized in Table 1. The complete product profile, including glucose (*C*1), cellotriose (*C*3), cellotetraose (*C*4), cellopentaose (*C*5), as well as the % cellulose conversion, is described in Additional file 1: Table S1. According to the results, most of the enzymes produce significant amounts of COS from all three substrates, while the production of glucose (if any) is relatively low. No product release (DP 1–5) was detected by the activity of *Cc*Cel9J, whereas the total conversion of cellulose into soluble oligosaccharides was higher in the case of *Ct*Cbh5A, *Pa*Cbh6A and *Cc*Cel9A.

**Table 1:** Relative proportion of cellobiose out of the total products released from the hydrolysis of CMC, PASC and Avicel and calculated degree of processivity of various cellulases used in this study

Enzyme name	CMC		PASC		Avicel	
	% cellobiose	Degree of processivity (*P*)	% cellobiose	Degree of processivity (*P*)	% cellobiose	Degree of processivity (*P*)
1. *Cc*Cel48A	49.4	1.9	57.2	2.5	61.6	1.9
2. *Ct*Cbh5A	78.3	4.7	71.1	2.4	79	3.6
3. *Ct*Cel9A	33.7	1.8	47.7	1.4	52.2	2.2
4. *Ct*Cbh9A	44.8	0.8	0	0.7	0	–
5. *Ct*Cel9B	70.8	2.6	75.4	3.8	70.1	2.3
6. *Pa*Cbh6A	85.9	5.2	87.2	7.0	92.5	12.0
7. *Cc*Cel9W	53.6	1.1	40.2	0.8	50.7	0.9
8. *Cc*Cel9M	25.2	0.8	30	0.5	36	0.4
9. *Cc*Cel9R	14.8	0.4	16	0.6	30	0.5
10. *Cc*Cel9A	81.4	3.8	80.7	3.7	85.2	5.5
11. *Cs*Cbh48A	59.5	2.1	70.2	3.1	75.8	3
12. *Cc*Cel9J	0	–	0	–	0	–
13. *Cc*Cel9Q	59	1.8	60.1	1.6	74	2.8
14. *Rf*Cel9A	44.2	1.9	35.5	1.6	56.9	2.7

The estimation of degree of processivity for each enzyme, also shown in Table 1, was based on the release of *C*2 and *C*3 products. *Pa*Cbh6A, *Cc*Cel9A and *Ct*Cbh5A exhibited the highest selectivity among other enzymes, with *Pa*Cbh6A and *Cc*Cel9A to release higher amounts of *C*2 as the substrate crystallinity increases. More specifically, the fungal *Pa*Cbh6A showed a high degree of processivity that was approximately 2.5 times higher on Avicel rather than amorphous CMC. These three biocatalysts were first selected as the most promising enzymes to be included in the enzyme cocktail for the production of cellobiose from lignocellulosic materials. *Cc*Cel48A, *Ct*Cel9A, *Ct*Cbh9A, *Cc*Cel9M, *Cc*Cel9R, *Cs*Cbh48A, *Cc*Cel9Q and *Rf*Cel9A did not produce only cellobiose but also *C*3–*C*5 products, thus suggesting that they performed random cleavages along the cellulosic molecule. Others, like *Ct*Cbh5A, *Ct*Cel9B, *Pa*Cbh6A, *Cc*Cel9W and *Cc*Cel9A, are recognized as enzymes with exo-activity, as they release *C*2 and *C*3 with no presence of C4 or C5. Moreover, they exhibit the higher degree of processivity among other enzymes tested, except for *Cc*Cel9W.

The results of the activity tests of the enzymes on organosolv-pretreated birch and spruce are shown in Table 2 and Additional file 1: Table S2. The results regarding the total product profile and the relative proportion of cellobiose released are similar to those observed for the pure cellulosic substrates for enzymes *Ct*Cbh5A, *Ct*Cel9B, *Pa*Cbh6A, *Cc*Cel9W, *Cc*Cel9M, *Cc*Cel9J, *Cc*Cel9Q*. Ct*Cbh5A, *Pa*Cbh6A, *Cc*Cel9M, *Cc*Cel9A exhibit the highest activity on birch (both B1 and B2) and spruce, while C*s*Cbh48A also releases a high amount of oligosaccharides from spruce. *CtCel9A* and *Rf*Cel9A were active only on spruce. C*c*Cel48A exhibits a sharp increase in both cellobiose yield and the degree of processivity value. *Ct*Cel9A and *Ct*Cbh9A do not seem active on these substrates, while *Cc*Cel9M and *Cs*Cbh48A release much lower amount of *C*2 than in pure substrates. The different specificity and mode of action regarding the release of oligosaccharides can be attributed to the different crystallinity and overall properties of the substrates. The degree of processivity value for the enzymes *Cc*Cel9W, *Cs*Cbh48A and *Cc*Cel9Q is higher when acting on lignocellulosic biomass compared to the CMC, PASC and Avicel.

**Table 2:** Relative proportion of cellobiose out of the total products released from the hydrolysis of lignocellulosic biomass and calculated degree of processivity of enzymes

Enzyme name	Birch (B1)		Birch (B2)		Spruce (S1)	
	% cellobiose	Degree of processivity (*P*)	% cellobiose	Degree of processivity (*P*)	% cellobiose	Degree of processivity (*P*)
1. *Cc*Cel48A	90.9	10	85	14.2	73.6	4.7
2. *Ct*Cbh5A	77.6	3	78.5	2.8	81	3.4
3. *Ct*Cel9A	0	–	0	–	58	1.9
4. *Ct*Cbh9A	0	0	1.5	0	0	0
5. *Ct*Cel9B	76.6	3.3	71.2	2.4	59.6	2
6. *Pa*Cbh6A	94.9	18.6	92.3	11.1	92.1	11.7
7. *Cc*Cel9W	67.2	4	79.6	6.1	63.4	2.3
8. *Cc*Cel9M	41.1	0.1	61.4	0.8	42.2	0.2
9. *Cc*Cel9R	82.9	6.6	79.5	6.9	26.6	1
10. *Cc*Cel9A	80.2	4.3	84.6	5.1	86.8	5.8
11. *Cs*Cbh48A	87.4	6.7	87.8	8.1	86.2	5.9
12. *Cc*Cel9J	0	–	0	–	0	–
13. *Cc*Cel9Q	63.8	3.3	73.8	4.2	77.2	2.9
14. *Rf*Cel9A	0	–	0	–	53.9	3.5

#### ii. Kinetics of hydrolysis of oligosaccharides

In order to estimate the catalytic efficiency of the processive enzymes on the soluble oligosaccharides with DP ranging from 5 to 8, a kinetics study was performed, and the catalytic efficiency *k*_cat_/*K*_m_ was calculated. According to the results depicted in Table 3, different activity and cleavage pattern of each enzyme are noticed as the polymerization degree (DP) increases. Particularly, there is a class of enzymes that the catalytic efficiency is increased together with the increase in the oligosaccharide DP (C*c*Cel48A, C*t*Cbh5A, P*a*Cbh6A, C*c*Cel9W, C*s*Cbh48A, C*c*Cel9Q, R*f*Cel9A), while other enzymes show decreased activity as the number of glucose units increases (C*t*Cel9A, C*t*Cbh9A, C*c*Cel9R). On the other hand, there are also some enzymes that their activity remains same despite the increase in the DP (C*t*Cel9B, C*c*Cel9A). The length of the oligosaccharides released from the activity of *Cc*Cel48A shows that the enzyme is very active on *C*7 and *C*8, and, after the first cleavage (releases *C*2 or *C*3), it prefers to cleave another *C*8 molecule rather than further cleave *C*5 to *C*2 and *C*3. *Ct*Cbh5A, *Cc*Cel9W, *Cs*Cbh48A, *Cc*Cel9Q and *Rf*Cel9A exhibit similar catalytic efficiency on oligosaccharides with DP 5–7, while it is much higher for *C*8. *Pa*Cbh6A prefers *C*7 and *C*8 rather than *C*5 and *C*6, while *Ct*Cel9A, *Ct*Cbh9A and *Cc*Cel9R have very low activity on *C*7 and *C*8. C*t*Cbh5A, P*a*Cbh6A, *Cc*Cel9W and *Cc*Cel9M showed the highest catalytic efficiency and were those that also exhibit the highest activity yields on the polysaccharidic substrates.

**Table 3:** Estimation of *k*_cat_/*K*_m_ and product profile of the processive cellulases on cello-oligosaccharides with DP 5–8

Enzyme name	*k*cat/*K*m (min^−1^ M^−1^)				Mode of action^a^			
	*C*5	*C*6	*C*7	*C*8	*C*5	*C*6	*C*7	*C*8
1. *Cc*Cel48A	1.37 × 10^3^	2.89 × 10^3^	4.35 × 10^3^	4.41 × 10^3^	*C*3 + *C*2	*C*4 to *C*2	*C*4 to *C*1	*C*5 to *C*1
2. *Ct*Cbh5A	8.18 × 10^3^	8.15 × 10^3^	8.3 × 10^3^	9.01 × 10^3^	*C*3 + *C*2	*C*4 to *C*2	*C*4 to *C*2	*C*6 to *C*1
3. *Ct*Cel9A	6.45 × 10^2^	6.76 × 10^2^	2.55 × 10^2^	9.3 × 10^1^	*C*3 + *C*2	*C*4 to *C*2	*C*4 to *C*2	*C*5 to *C*2
4. *Ct*Cbh9A	7.46 × 10^2^	7.61 × 10^2^	2.01 × 10^2^	2.36 × 10^2^	*C*4 to *C*1	*C*4 to *C*2	*C*4 to *C*2	*C*5 to *C*2
5. *Ct*Cel9B	1.71 × 10^3^	1.56 × 10^3^	1.59 × 10^3^	1.88 × 10^3^	*C*3 + *C*2	*C*4 to *C*2	*C*5 to *C*2	*C*6 to *C*2
6. *Pa*Cbh6A	5.13 × 10^3^	3.46 × 10^4^	8.87 × 10^4^	8.11 × 10^4^	*C*3 + *C*2	*C*4 to *C*2	*C*4 to *C*2	*C*5 to *C*2
7. *Cc*Cel9W	1.13 × 10^3^	8.74 × 10^3^	2.18 × 10^4^	2.86 × 10^4^	*C*3 + *C*2	*C*4 to *C*2	*C*4 to *C*2	*C*5 to *C*2
8. *Cc*Cel9M	2.99 × 10^4^	2.52 × 10^4^	3.17 × 10^4^	2.78 × 10^4^	*C*4 + C1	*C*4 + C2	*C*4 to *C*2	*C*5 to *C*2
9. *Cc*Cel9R	–	2.04 × 10^3^	1.23 × 10^3^	8.51 × 10^2^	–	*C*3	*C*4 + *C*3	*C*5 to *C*3
10. *Cc*Cel9A	1.37 × 10^3^	1.34 × 10^3^	1.34 × 10^3^	1.79 × 10^3^	*C*4 to *C*1	*C*4 to *C*2	*C*5 to *C*2	*C*4 to *C*2
11. *Cs*Cbh48A	1.00 × 10^3^	1.56 × 10^3^	2.97 × 10^3^	3.70 × 10^3^	*C*3 + *C*2	*C*4 to *C*2	*C*6 to *C*1	*C*6 to *C*1
12. *Cc*Cel9J	–	–	–	–	–	–	–	–
13. *Cc*Cel9Q	3.26 × 10^3^	3.81 × 10^3^	5.04 × 10^3^	6.51 × 10^3^	*C*3 + *C*2	*C*4 to *C*2	*C*4 to *C*2	*C*6 to *C*2
14. *Rf*Cel9A	1.12 × 10^2^	1.94 × 10^3^	5.77 × 10^3^	5.93 × 10^3^	–	*C*4 to *C*2	*C*5 to *C*2	*C*6 to *C*2

^a^“*C*2 + C3” represents the products *C*2 and *C*3, while “*C*4 to *C*1” represents a range of products with degree of polymerization varying between 4 and 1, i.e., from cellotetraose to glucose (*C*4 + *C*3 + *C*2 + *C*1)

Summarizing all the results from the activity tests on cellulosic polysaccharides (CMC, PASC, Avicel), oligosaccharides and lignocellulosic materials, the enzymes C*t*Cbh5A and P*a*Cbh6a best satisfy the requirements for high activity and degree of processivity, high cellobiose production and low glucose production. Thus, these enzymes were chosen as the most suitable for the construction of an enzymatic cocktail together with the other key enzymes for cellobiose production. C*t*Cbh5A, when added to the cocktail, rather decreased the cellobiose yield instead of acting synergistically with other processive enzymes (data not shown), and therefore, it was not included in any further study. On the contrary, the activity tests that were performed for the P*a*Cbh6A showed that when this enzyme was added to a cocktail, the cellobiose yield increased. As a result, P*a*Cbh6A was considered as the most promising to be included in the experimental design for birch and spruce biomass hydrolysis in order to determine the optimal enzyme combination for the maximum cellobiose yield.

### Optimization experiments and construction of a defined enzyme cocktail

Preliminary experiments were run in order to identify the upper and lower limits for each enzyme relative proportion in the cocktail. The preliminary results with the recombinant enzymes enabled the selection of the appropriate limits of the relative abundances of the enzymes for the experimental design. The results, as depicted in Table 4, showed that for all the lignocellulosic substrates the optimum combination of the enzymes that maximizes the cellobiose yield is same and particularly the combination #3, which corresponds to 30% *Tt*EG5, 30% *Tt*LPMO and 40% *Tt*CBH7. This observation confirms that a relative abundance of all the three enzymes is needed and thus the construction of an enzymatic cocktail provides higher cellobiose yields. The cellobiose yield that is achieved by this enzyme combination for birch is 10.3% and 13.0% (% w/w cellulose conversion into cellobiose) for B1 and B2, respectively. For spruce, the maximum cellobiose yield that is achieved is 8.4%. It can be therefore noticed that B2 gives higher cellobiose yields among the other lignocellulosic substrates, leading to the production of 90.4 mg of cellobiose/g of substrate. In general, spruce gives slightly lower hydrolysis yields which can be attributed to the higher lignin content (14. 9%) compared to birch (B1: 7.8%, B4: 7.1%). Additionally, it should be mentioned that the cellobiose-to-glucose ratio is quite low for B2 compared to B1, while spruce exhibits much higher ratio.

**Table 4:** Hydrolysis data from preliminary experiments with EG5, *Mt*CBH7 and *Tt*LPMO

Run	EG5	*Tt*LPMO	*Mt*CBH7	*C*2%	*C*1%	*C*2%	*C*1%	*C*2%	*C*1%	*C*2/*C*1		
	%	%	%	Β1		Β2		S1		B1	B2	S1
1	1	0	0	5.97	0.32	4.69	0.94	5.7	0.28	18.8	5	20.4
2	0	0	1	5.98	1.05	7.87	2.58	4.15	0.29	5.7	3	14.3
3	0.3	0.3	0.4	10.3	1.58	13.02	2.62	8.39	0.44	6.5	5	18.9
4	0.4	0.1	0.5	9.91	1.38	11.16	2.51	5.64	0.32	7.2	4.5	17.5
5	0.3	0.1	0.6	7.41	1.43	12.01	2.64	4.75	0.32	5.2	4.6	14.8
6	0.5	0	0.5	8.68	1.55	10.12	2.44	4.89	0.28	5.6	4.1	17.3
7	0	0.2	0.8	9.06	1.86	10.1	2.75	4.47	0.34	4.9	3.7	13.3
8	0	0.3	0.7	8.07	1.38	7.69	2.08	3.49	0.26	5.9	3.7	13.2
9	0.8	0.2	0	6.35	0.52	5.98	1.14	5.39	0.29	12.3	5.3	18.3
10	0.7	0.3	0	5.63	0.38	6.13	1.25	6.33	0.36	15	4.9	17.5

The processive enzyme that was selected to be included in the cocktail, *Pa*Cbh6A, was tested in different relative proportions together with EG5, *Tt*CBH7, *Tt*LPMO and *Tt*EG7. The four core enzymes (EG5, CBH7, *Tt*LPMO and *Pa*Cbh6A) participated in the 95% of the enzyme mixture, while EG7 consisted 5% of the enzyme mixture and was added on a fixed proportion in all experimental runs. The % w/w cellulose conversion into cellobiose was used as the response factor to estimate the hydrolysis rate. All data are summarized in Additional file 1: Table S3. In the case of birch, the hydrolysis data for 24 h of reaction were fitted to a quadratic model (*R*^2^ = 0.7404, *p* = 0.0434); a similar model was also used for the case of 48 h of hydrolysis (*R*^2^ = 0.8071, *p* value = 0.0124) (Additional file 1: Table S4). Optimization prediction targeting the maximal release of cellobiose for both 24 and 48 h generated a ternary enzyme mixture comprised of 20% EG5, 43% CBH7, 22% *Tt*LPMO, 10% *Pa*Cbh6a and 5% EG7. The theoretical cellobiose yield was predicted to be 21.86% w/w cellulose conversion. As depicted in the ternary plots in Additional file 1: Figure S1, CBH7 is the key enzyme for hydrolysis for both early and late stages of reaction, while the contribution of LPMO and EG5 is of pivotal importance. Addition of *Pa*Cbh6A and EG7 at lower percentage favors the release of cellobiose, by comparing the results with the preliminary results in Table 4. The cellobiose yield that was predicted from the optimal combination was verified experimentally. It was shown that this enzyme mixture leaded to 22.3% w/w cellulose conversion to cellobiose; this value was slightly higher than the one predicted.

Similarly, for the hydrolysis of spruce biomass, quadratic model was used for analyzing the data after 24 h (*R*^2^ = 0.9072, *p* value = 0.0004) and 48 h of hydrolysis (*R*^2^ 0.8711, *p* value = 0.0008) (Additional file 1: Table S4). Optimization prediction generated a ternary enzyme mixture comprised of 35% EG5, 45% CBH7, 10% *Tt*LPMO, 10% P*a*Cbh6a and 5% EG7, with a theoretical yield of 19.6% w/w cellulose conversion into cellobiose. CBH7 and EG5 activities are crucial for the hydrolysis, as shown as these enzymes constitute 75% of the total mixture. The plots at Additional file 1: Figure S2 reveal that EG5 is necessary at the initial stage of hydrolysis (according to data at 24 h), while CBH7 is the key enzyme for maximizing cellobiose at 48 h. The experimental yield of the optimal combination was 19.1% w/w cellulose conversion. After optimization and experimental verification of their performance, the optimal enzyme mixtures that maximize the cellobiose production were employed for the scale-up reaction and production of COS.

### Nanofiltration studies

In order to choose the best membrane to remove glucose from the hydrolysate, screening of five different nanofiltration membranes was performed. The effect of different parameters was tested for evaluating the performance of each membrane regarding the best separation of cellobiose/glucose.

#### i. Water permeability

The average water permeabilities (*L*_p_) of all the membranes were calculated with Eq. 5 and are depicted in Additional file 1: Table S5. The loss of initial water permeability during the nanofiltration trials was found to be 1 unit for all the membranes (data not shown). Hence, membrane fouling did not seem significant in any of the cases.

#### ii. Effect of feed concentration

The separation factor for the different membrane systems, given by Eq. 7, is depicted in Table 5. The pure sugars model solution comprised of cellobiose and glucose was tested at different feed concentrations, with a constant molar ratio of cellobiose to glucose equal to 9:1. An increase in the total feed concentration resulted, as expected, in reduced permeate flux (data not shown), as a result of the higher osmotic pressure of the solution. However, increasing the feed concentration did not affect significantly the cellobiose separation factor for all the different pressure conditions applied in this study. In fact, only a slight decrease in the separation factor (within the range of 0.01–0.07) was noticed, which can be attributed to the increase in the total sugar amount. The feed concentration did not exhibit a significant effect neither on the % retention of cellobiose or on glucose. Only in the case of DL and NF270 membranes, a minor increase in the retention was observed, following the increase in the feed concentration, at the range of 2.4% for cellobiose (DL membrane) and 6.6% for glucose (NF270 membrane). In the case of the NFX membrane, the trials were carried out only at 5 bar, as the permeate flux was very high (data not shown) and the separation factor was the lowest (1.00); therefore, this system was not chosen to be used in any further study.

**Table 5:** Separation factors and sugar retention for all nanofiltration membranes at different feed concentrations of the pure sugar mixture, in various pressure conditions

Membrane	Feed concentration (mg/mL)	Separation factor				Retention (% w/w)^a^	
		5 bar	10 bar	15 bar	20 bar	Cellobiose	Glucose
NF270	5	–	1.12	–	1.03	96.3	86.4
	20	–	1.05	–	1.02	97.4	93.0
DL	5	1.11	1.14	1.11	1.03	86.6	76.0
	20	–	1.10	1.07	1.07	89.0	81.3
NFX	5	–	1.00	–	–	98.8	98.5
NFW	5	1.01	–	–	–	64.0	63.2
	20	0.96	–	–	–	58.1	60.4
TS40	5	–	1.03	1.07	–	93.0	90.2
	20	–	1.11	1.04	–	93.6	84.4

^a^The retention of each sugar has been estimated for a pressure of 10 bar, apart from the NFX membrane that was tested only at 5 bar

#### iii. Effect of pressure

The effect of pressure on the cellobiose separation factor was studied. It was investigated whether an increase in the pressure applied in the nanofiltration vessel could affect the cellobiose/glucose separation factor. The overall conclusion comparing the separation factor values of Table 5 for all the membranes is that higher pressure leads to a decrease in the separation factor. Therefore, the optimal pressure conditions in order to achieve the maximum separation were set up at 10 bar. The membranes that appeared to perform the higher separation at 10 bar were the membrane DL (with a separation factor of 1.14), NF270 (1.12) and TS40 (1.11). For evaluating the membranes’ efficiency toward the enhanced separation of cellobiose/glucose system, a comparison of the observed retentions of cellobiose and glucose is necessary. Table 5 depicts the retention data, given by Eq. 6, that refer to the results for the trials that were carried out at room temperature and the optimized pressure of 10 bar, except from the NFX membrane where the applied pressure was 5 bar. An efficient separation is achieved by high retention of cellobiose and low retention for glucose. Thus, the membranes that best satisfy this demand are the membrane NF270 (96.3% for cellobiose and 86.4% for glucose) and the membrane TS40 (93.6% for cellobiose and 84.4% for glucose).

#### iv. Effect of temperature

The effect of different temperature conditions on the performance of the membranes that were selected (NF270, TS40) was investigated at 10 bar. Another pressure condition was also tested (20 bar) in order to investigate the possibility that temperature and pressure factors have a combined effect on the separation efficiency of the membranes. As shown in Table 6A, applying higher temperature is correlated with a more efficient separation process. The effect of temperature on the separation factor is more clearly presented when the feed concentration is equal to 20 mg/mL, since the separation factor is increased more significantly than in the case of 5 mg/mL feed concentration. When 20 mg/mL feed concentration was applied on the NF270 membrane, the separation factor increased at higher temperature conditions; however, the increase was mostly significant when the nanofiltration was performed at 40 °C (with a separation factor of 1.21), compared to that performed at room temperature (1.05), while it increased slightly at higher temperature (50 °C and 60 °C). When 20 mg/mL feed concentration was used on the TS40 membrane, the increase in the separation factor was significant when temperature increased from 40 °C (1.21) to 50 °C (1.39). Similar to the previous results, it can be noticed that application of higher pressure does not have a favorable effect on separation process; the increase in the separation factor was less significant at 20 bar rather than at 10 bar.

**Table 6:** (A) Separation factor for NF270 and TS40 membrane at different feed concentrations, pressure and temperature conditions. (B) Observed retention for cellobiose and glucose at different feed concentrations and temperatures with the model solution at 10 bar and 20 bar

(A)									
Membrane	Feed concentration (mg/mL)	Separation factor							
		10 bar			20 bar				
		40 °C	50 °C	60 °C	40 °C	50 °C	60 °C		
NF270	5	1.32	1.32	1.31	1.02	1.09	1.15		
	20	1.21	1.26	1.36	1.10	1.08	1.29		
TS40	5	1.00	1.16	–	–	–	–		
	20	1.21	1.39	–	–	–	–		

(B)									
Membrane	Feed concentration (mg/mL)	Retention^a^ (%)							
		RT		40 °C		50 °C		60 °C	
		*C*2	*C*1	*C*2	*C*1	*C*2	*C*1	*C*2	*C*1
NF270	5	96.3	86.4	91.4	69.2	89.1	72.6	87.6	66.8
	20	97.4	93.0	95.5	79.1	91.2	72.5	94.9	69.9
TS40	5	93.0	90.2	99.3	99.2	98.6	84.6	–	–
	20	93.6	84.4	91.3	75.5	89.3	64.3	–	–

*RT* room temperature

^a^The retention of each sugar has been estimated for a pressure of 10 bar

However, according to the results depicted in Table 6B, it can be observed that as the temperature increases, the % retention for both cellobiose and glucose decreases. Consequently, the selection of the optimal temperature condition depends on both the membrane separation performance and the purpose of the filtration. If higher % cellobiose retention and elimination of sugar losses are targeted, then nanofiltration should be performed at room temperature (25 °C). If the aim of the study is to achieve the highest separation of the cellobiose/glucose mixture regardless of cellobiose losses, then increasing the operating temperature is suggested. In the case of NF270 membrane, the optimal temperature is 40 °C, while TS40 performs the optimum separation of cellobiose and glucose at 50 °C.

#### v. Nanofiltration with the enzymatic hydrolysate

A screening of the membranes was performed with the birch hydrolysate solution in order to evaluate the performance of the membranes toward the separation of cellobiose and glucose and compare the results with those in the case of the pure sugars model solution. The trials were carried out at the optimal pressure condition of 10 bar and at room temperature for all the membranes except for the membrane NFW which showed very low performance on the previous trials from the trials with the model solution. For the membranes that proved to be more efficient (NF270 and TS40), the trials were also carried out at higher temperatures (40, 50 and 60 °C). As shown in Table 7, the results are in accordance with those observed in trials with the pure sugars model solution. Particularly, the membranes with the highest separation factor were NF270 (1.58 at 40 °C) and TS40 (1.35 at 50 °C). From the results in Table 7 it can be observed that the retention for both cellobiose and glucose reduced with the increase in the temperature for membrane NF270, while for TS40 the cellobiose retention increased from 40 °C (91.2%) to 50 °C (94.6%). Similar conclusions are made regarding the most efficient separation of cellobiose/glucose as before for the pure sugar mixture. If higher cellobiose retention is required, the optimal operating temperature is 25 °C, while for achieving the highest separation, the optimal temperature is 40 °C for membrane NF270 and 50 °C for membrane TS40.

**Table 7:** (A) Separation factor and (B) sugar retention for different membranes and temperature conditions with the enzymatic hydrolysate at 10 bar

(A)								
Membrane	Separation factor							
	RT	40 °C	50 °C	60 °C				
NF270	1.06	1.58	1.26	1.32				
DL	1.04	–	–	–				
NFX	1.01	–	–	–				
TS40	–	1.13	1.35	–				

(B)								
Membrane	Retention^a^ (%)							
	RT		40 °C		50 °C		60 °C	
	*C*2	*C*1	*C*2	*C*1	*C*2	*C*1	*C*2	*C*1
NF270	98.3	92.8	88.3	55.9	88.9	70.5	83.9	63.5
DL	88.2	84.6	–	–	–	–	–	–
NFX	99.8	98.4	–	–	–	–	–	–
TS40	–	–	91.2	80.9	94.6	69.9	–	–

^a^The retention of each sugar has been estimated for a pressure of 10 bar

### Scale-up reaction and product recovery

**Fig. 1:**
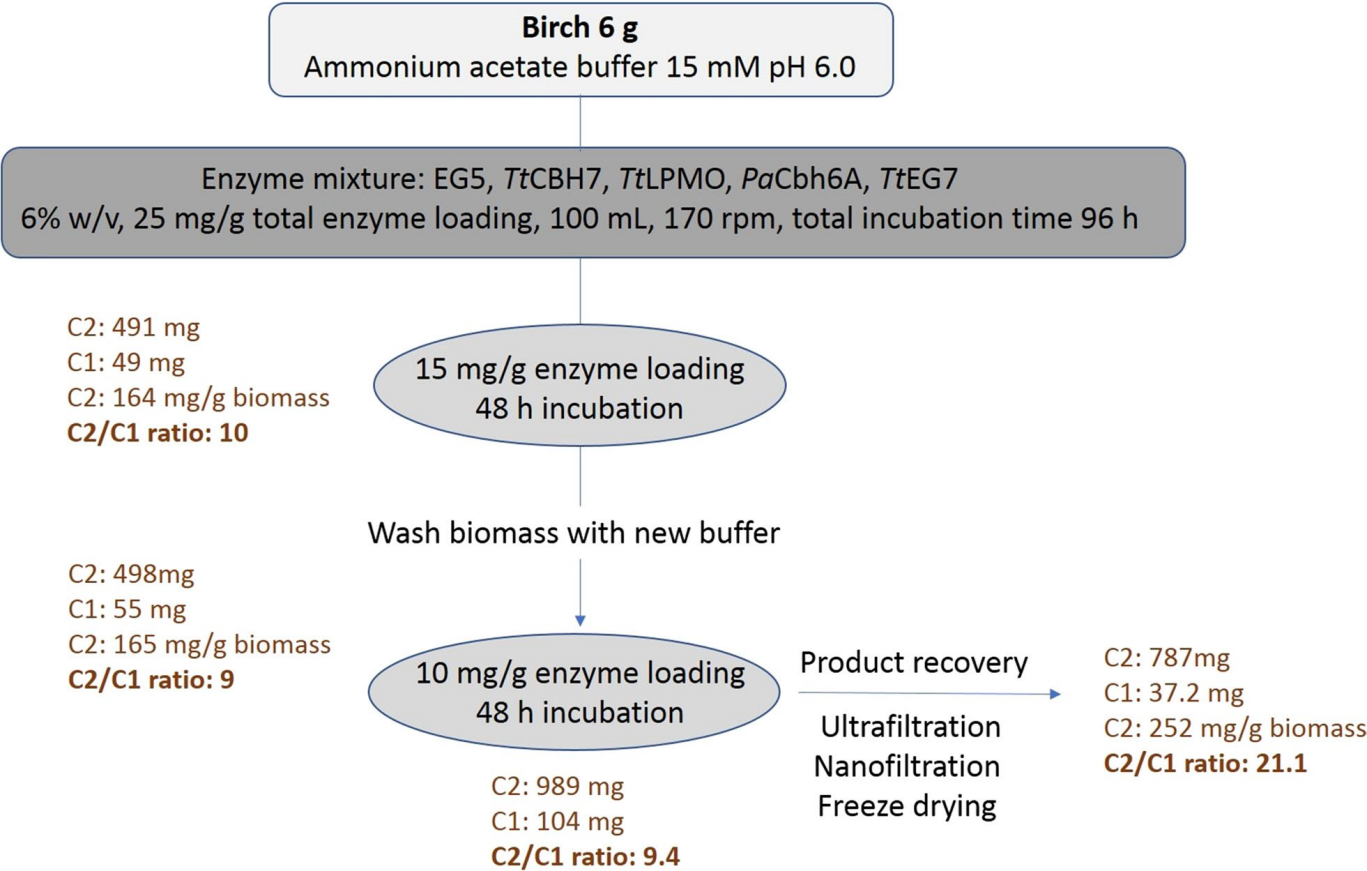
Overall scheme of the cellobiose production from pretreated birch

A scale-up reaction with a total volume of 100 mL was carried out using the optimized enzymatic combinations that were determined for each substrate (birch B1 and spruce S2). The main target was the maximum production of cellobiose, the concentration and product recovery out of the hydrolysate and, finally, the tests as a carbohydrate source for several probiotic strains. TFF and nanofiltration method was applied as a product recovery method, even though during the filtration, a slight amount of cellobiose amounts can be removed in the permeate. Figure 1 represents the overall procedure for the production of cellobiose from birch, as well as the product yield and recovery in each stage. A total amount of 989 mg of cellobiose was produced from the enzymatic hydrolysis of 6 g of initial biomass, corresponding to 164 mg of cellobiose/g of substrate, while after filtration, 787 mg of the final product remained. The nanofiltration step resulted in the removal of a great amount of glucose, leading to a final cellobiose-to-glucose ratio of 21.1. In the case of spruce, the enzymatic hydrolysis yielded 128 cellobiose/g of substrate with a 17.4 cellobiose: glucose ratio, while after nanofiltration, the final product was comprised of 651 mg cellobiose and 32 mg glucose (20.2 cellobiose: glucose ratio). The products were freeze-dried and used for evaluation of their prebiotic potential.

### Evaluation of COS prebiotic activity

#### i. Growth potential of *Bifidobacteria* and *Lactobacilli* strains on pure cellobiose

**Fig. 2:**
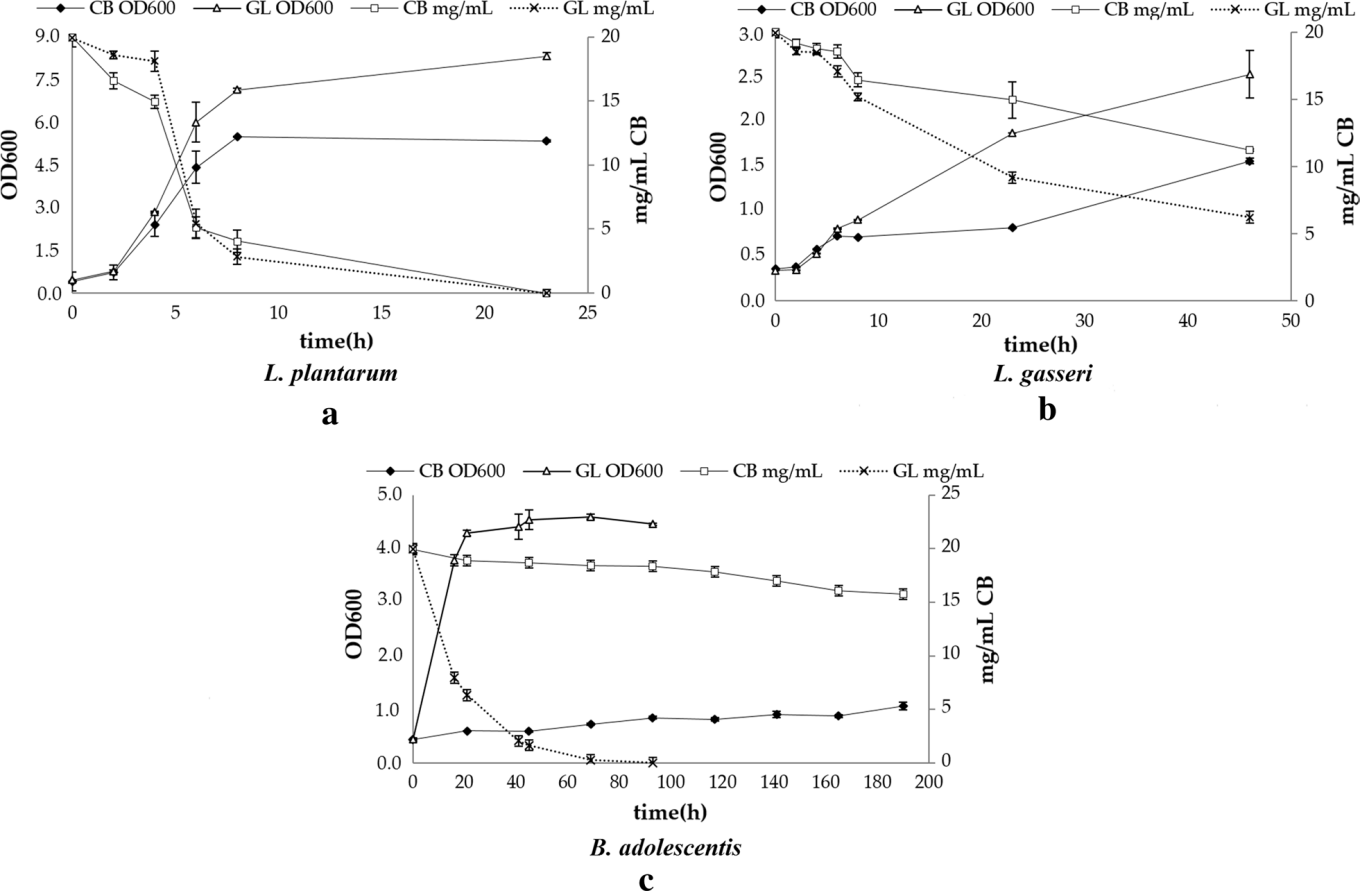
Growth curve and carbohydrate consumption of **a** *L. plantarum*, **b** *L. gasseri* and **c** *B. adolescentis* grown on culture media supplemented with 2% (w/v) cellobiose and 2% (w/v) glucose

The ability of different bacterial probiotic strains to utilize pure cellobiose as carbon source was studied and compared to their growth rate on glucose. The results, as evaluated by the increase in the optical density (OD_600_) and the carbohydrate accumulation, are summarized in Table 8 and depicted in Fig. 2. Cultivation in the absence of any carbon substrate did not show any growth of the probiotic strains. Only one of the *Bifidobacteria* strains, namely *B. adolescentis*, showed a slight growth on culture media supplemented with 2% w/v cellobiose. The growth rate of *B. adolescentis* on cellobiose was *μ* = 0.014 h^−1^ which was considered very low compared to that when grown on glucose (*μ* = 0.107 h^−1^), also verified by the low value of the final optical density of the culture (OD_600_ = 1.06 ± 0.07). Therefore, this strain was not chosen for further studies to evaluate the prebiotic potential of lignocellulosic-derived COS. Among the *Lactobacilli* strains, two of them (*L. gasseri* and *L. plantarum*) could efficiently grow on cellobiose. *L. gasseri* strain can utilize both carbon sources which is demonstrated by the similar growth rates in cellobiose and glucose (*μ* = 0.212 h^−1^) and exhibits a relatively high growth in cellobiose (final OD_600_ = 1.56 ± 0.03). *L. plantarum* appeared to be the most promising probiotic strain for further studies, as it shows the highest growth rate in cellobiose (*μ* = 0.407 h^−1^) and a final OD_600_ = 5.35 ± 0.04. Moreover, in this study, it was the only strain that consumed the total carbohydrate content within the first 25 h of fermentation, as shown in Fig. 2c). The growth rates of all the strains were lower when cultured in cellobiose than when cultured in glucose. Lactic acid is the only metabolite that is produced by both *L. gasseri* and *L. plantarum* when grown on cellobiose, as depicted in Table 9, while no production of any short chain fatty acid (acetic, propionic, butyric acid) was detected.

**Table 8:** Cellobiose utilization by *Bifidobacterium* and *Lactobacillus* strains

*Bifidobacteria* strains	Cellobiose growth	*Lactobacilli* strains	Cellobiose growth
*B. adolescentis* DSM 20083	+	*L. gasseri* DSM 20077	++
*B. longum* DSM 20219	−	*L. plantarum* ATCC 8014	++++
*B. animalis* subsp. lactis	−	*L. reuteri* DSM 20016	−

A minus sign (−) indicates that final OD_600_ < 0.6, + indicates final OD_600_ = 0.6–1, ++ indicates final OD_600_ = 1–2, +++ indicates final OD_600_ = 2–5, and ++++ indicates final OD_600_ > 5

**Table 9:** Fermentation metabolites of (A) *L. gasseri* and (B) *L. plantarum* upon growth on pure cellobiose, birch and spruce COS-rich hydrolysates

(A)						
	*L. gasseri*					
	Cellobiose		Birch		Spruce	
	0 h	26 h	0 h	26 h	0 h	95 h
Cellobiose	20.0 ± 1.06	9.3 ± 0.12	19.6 ± 2.04	0.0 ± 0.00	19.3 ± 0.06	19.2 ± 0.29
Lactic acid	0.33 ± 0.06	8.53 ± 0.91	0.2 ± 0.03	26.5 ± 1.52	0.2 ± 0.09	0.5 ± 0.20
Acetic acid	4.03 ± 0.21	4.07 ± 0.05	7.9 ± 1.04	8.0 ± 0.42	6.6 ± 0.19	7.2 ± 0.12
Propionic acid	0.93 ± 0.03	1.03 ± 0.12	0.8 ± 0.10	1.3 ± 0.11	0.7 ± 0.15	0.8 ± 0.01

(B)						
	*L. plantarum*					
	Cellobiose		Birch		Spruce	
	0 h	26 h	0 h	26 h	0 h	95 h
Cellobiose	20.0 ± 1.51	0.2 ± 0.00	19.8 ± 1.69	0.0 ± 0.00	19.9 ± 0.76	10.8 ± 1.25
Lactic acid	0.4 ± 0.01	21.8 ± 2.85	0.3 ± 0.05	28.0 ± 2.67	0.3 ± 0.00	21.1 ± 1.67
Acetic acid	4.2 ± 0.15	3.9 ± 0.71	7.9 ± 0.92	8.4 ± 0.91	6.9 ± 0.25	8.8 ± 1.61
Propionic acid	0.9 ± 0.01	0.9 ± 0.03	0.8 ± 0.09	1.0 ± 0.04	0.6 ± 0.02	1.2 ± 0.15

No significant amounts of formic or butyric acid were detected

#### ii. Growth potential of *Lactobacillus* strains on plant-derived COS

**Fig. 3:**
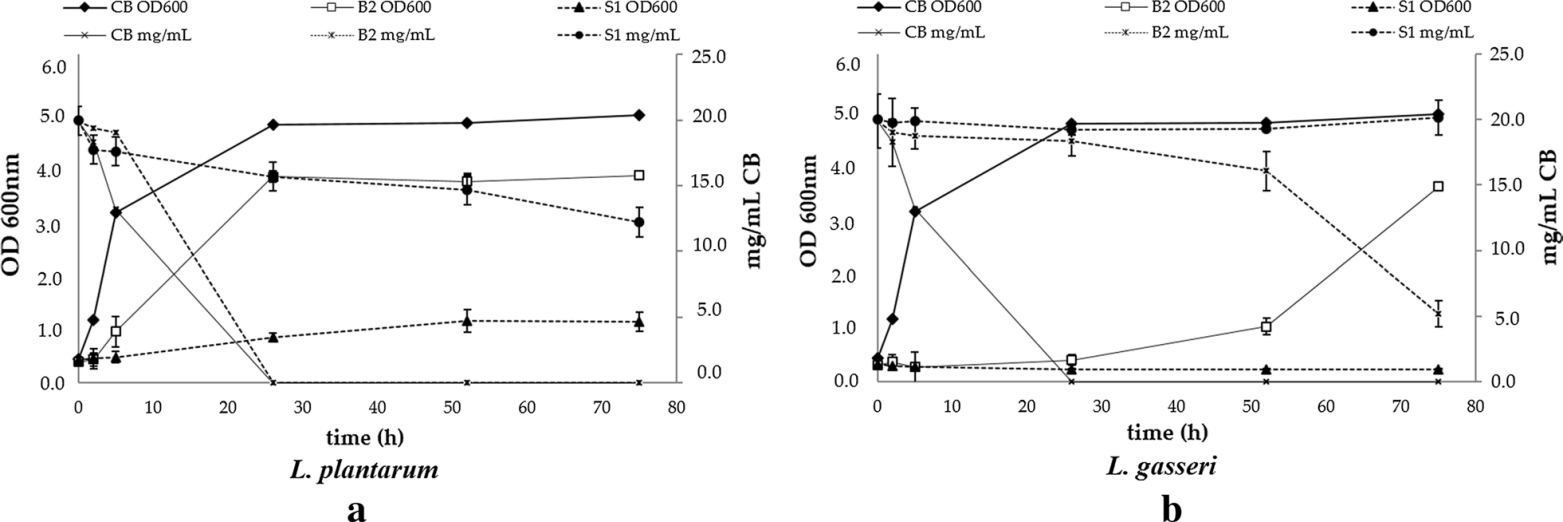
Growth curve and carbohydrate consumption of **a** *L. plantarum* and **b** *L. gasseri* grown on culture media supplemented with biomass hydrolysates at an initial concentration of 2% (w/v) cellobiose

The *Lactobacillus* strains that were able to utilize pure cellobiose as carbon source were used for further testing the prebiotic effect of the plant-derived cello-oligosaccharides (COS). The results showed that both strains are able to grow on birch-derived COS as shown in Fig. 3 by the increase in the optical density value and the consumption of the cellobiose content. The most effective strain was *L. plantarum*, with a growth rate of *μ* = 0.161 h^−1^, while *L. gasseri* could also utilize this carbon source and grow. In the case of spruce-derived sugars, *L. plantarum* strain exhibited very slow growth (*μ* = 0.039 h^−1^), while *L. gasseri* did not grow at all. Determination of the fermentation products (Table 9) reveals the presence of other sugars existing in the biomass hydrolysate that were not detected by HPLC and can be consumed by *L. plantarum*, since the amount of the lactic acid produced is much higher than the cellobiose that is consumed. In fact, a low amount of glucose is present, but still the final concentration of lactic acid is much higher. Analysis of hydrolysates with HPAEC-PAD chromatography revealed traces of cellotriose, cellotetraose, as well as some oxidized products; however, it is possible that oligosaccharides with higher DP exist in the hydrolysate and are consumed by the bacteria. The same was also observed for the growth of *L. gasseri* on birch hydrolysate. In a similar way as for the cellobiose substrate, no production of acids was observed. The acetic acid detected at the beginning of the fermentation was originated from the cultivation media and the buffer that was used in the hydrolysis step and was not removed during nanofiltration process.

## Discussion

Forest residues are by nature heterogeneous in composition, size, structure and properties. Thus, the degradability of these materials is different. Birch stem wood contains about 43.9% w/w cellulose, while in the case of spruce this percentage is about 42% w/w [18]. To accomplish the efficient hydrolysis process of different types of lignocellulosic materials, novel lignocellulolytic enzyme mixtures have to be customized. The key enzymes leading to cellobiose production are cellobiohydrolases and processive endoglucanases. The degree of processivity is an estimation of the cleavage per productive binding of the enzyme onto the substrate. Processive enzymes release cellobiose. Throughout the literature, estimation of processivity can be problematic, although we assume that many enzymes will maximize the cellobiose release as a proof of their processive function. Assuming that the first product of an initial cleavage will be either *C*2 or *C*3, depending on the configuration of the cellulose molecule in the active site of the enzyme, processive enzymes will perform several cleavages along the same molecule, producing cellobiose as a soluble product, so the ratio of *C*2 to *C*3 will increase [18]. The mechanism for processivity has been correlated with structural properties and molecular characteristics of the enzyme, including the interactions of carbohydrate-binding module (CBM) with the substrate [19, 20]. Moreover, processivity is a rather substrate-specific feature of the enzyme and it differs along with the substrate properties, as it was shown from the results of this study. Some enzymes exhibited a higher degree of processivity on CMC, while others on the highly crystalline Avicel. P*a*Cbh6a showed a 2.5-times higher processivity on Avicel compared to CMC, which has also been reported in the literature for other fungal enzymes [21]. A shift between endo-activity and exo-activity depending on the substrate crystallinity has also been proposed [22].

Screening of different processive cellulases enabled the selection of some promising candidates which were then tested in enzyme cocktails together with processive endoglucanases and cellobiohydrolases for their ability to yield cellobiose. The results showed that CBH7 and EG5 were the key enzymes for the hydrolysis, but the addition of LPMO and CBH6 increased of product release in a cooperative action of the enzymes that act synergistically. It was found that the efficient hydrolysis of spruce requires a higher amount of CBH7 and EG5, which has also been reported in the literature [13]. CBH7s are the main catalysts for the production of cellobiose, but severe end-product inhibitory phenomena can occur in their active site; the addition of processive EGs with a wider active site area that are not so susceptible to cellobiose inhibition can boost the hydrolysis and maximize the product release [23].

Scaling-up of the process is always challenging and requires different strategies. The overall yield of the reaction is quite low, but it should be taken into consideration that this is a controlled hydrolysis and there is much of the end-product inhibition, as in nature, microorganisms have been evolved to secrete cocktails that are optimized to perform the complete hydrolysis of the substrate yielding glucose to be used as a carbon source. However, by altering the conditions of the reaction, like multistage hydrolysis together with enzyme recovery and re-usage, we can make the process economically viable, since food-grade prebiotic oligosaccharides can be produced by cheap and abundant biomass wastes.

The aim of the nanofiltration at the product purification step is the separation of cellobiose and glucose for obtaining a solution potentially pure of cellobiose. This step is especially important for enzymatically produced oligosaccharides. Nanofiltration is an easy maintenance and cost-competitive alternative that can offer promising results. However, cellobiose and glucose molar masses differ only by a factor of 1.9 which inevitably makes the separation process extremely difficult. Study of the effect of the different nanofiltration parameters, such as feed concentration and temperature, revealed that all values corresponding to the cellobiose separation factor are quite low (< 1.5), indicating that the cellobiose/glucose system is difficult to separate. An increase in the total feed concentration resulted, as expected, in reduced permeate flux (data not shown), as a result of the higher osmotic pressure of the solution [24]. Moreover, a slight decrease in the separation factor was observed, which can be attributed to the increase in the total sugar amount, which has been also mentioned before [25], even though that study was based on a different system (xylose/glucose). Regarding the effect of temperature, it has been mentioned in previous studies that an increase in the temperature affects the separation process by having an impact not only on the feed solution properties but also on the pore structure of the membrane [26]. By the increase in the temperature, the viscosity of the feed solution was decreased and the permeate flux increased. The latter can be explained by the pore swelling of the membrane [24]. As a result, nanofiltration with different membranes and different operating conditions can offer many possibilities depending on the application that is targeted, either high product recovery (100% of the initial cellobiose content) or high purity (low glucose yield). In our case, the selection of the most appropriate membrane was done following a stepwise strategy. Data from Table 5 of the revised manuscript show that NSF270 and DL membranes exhibit similar separation factors; however, NSF270 achieves a much higher % cellobiose retention. In order to prevent the losses, NSF270 was chosen for further studies. TS40 also presented a high % retention with relatively equal separation factor; therefore, this membrane was also tested on different temperature conditions. Further studies and comparison of TS40 and NSF270 showed that NSF270 outperformed TS40 regarding the separation factor; therefore, this membrane was selected for the scale-up reaction.

In this study, cultivations of six selected bacterial species, three *Lactobacilli* and three *Bifidobacteria* strains, using cello-oligosaccharides (COS) as carbon source showed selective growth of *Lactobacillus plantarum* ATCC 8014 and *Lactobacillus gasseri* DSM 20077. The efficient degradation of the forest biomass-derived COS by the *Lactobacilli* probiotic strains indicates a promising prebiotic potential. As noticed, COS from birch have positive effect for the probiotic strains, while those from spruce COS, on the other hand, did not prove to be an efficient carbohydrate substrate for *the Lactobacilli* strains. This can be possibly attributed to the higher lignin content of spruce and the release of products that can inhibit the growth of lactic acid bacteria after the enzymatic hydrolysis. It has been reported in the literature that the presence of phenolic compounds and furans, even in low amounts, can inhibit the growth of lactic acid bacteria [27]. The most promising probiotic strain for COS is the *L. plantarum* strain as it shows significantly higher growth rates and faster carbohydrate utilization than *L. gasseri* strain. The explanation for this is the strain’s largest genome size compared to other bacterial species and therefore its ability to encode higher number of phosphotransferase system genes [28, 29]. Thus, *L. plantarum* strains can ferment a wider range of carbohydrates.

## Conclusions

The main target of this study was to develop and optimize a novel process for the efficient production of cellobiose-rich hydrolysates from plant cell wall polysaccharides. These cello-oligosaccharides are produced by enzymatic hydrolysis processes using waste lignocellulosic biomass residues (birch and spruce) as substrates and are a very promising category of NDOs as they are originated by the most abundant carbon source, lignocellulose. The combination of physicochemical treatment and controlled enzymatic hydrolysis by employing a mixture of processive cellulases, together with nanofiltration for the recovery of the final product allowed the production of cellobiose from biomass. The potential of the hydrolysis products to support the in vitro growth of different *Lactobacilli* probiotic strains as a sole carbon source was demonstrated.

## Methods

### Enzymes and substrates

For the production of COS from biomass, different cellulases with processive activity were employed. Endo-1,4-β-d-glucanase (EG5) from *Talaromyces emersonii* was purchased from Megazyme (USA), while a total amount of 14 processive cellulases were purchased from NZYTech Lda. (Portugal) and are summarized in Table 10. Ten enzymes are classified in the glycoside hydrolase family 9 (GH9), two in GH48, one from GH6, and one from GH5 family. Apart from the commercially available enzymes, three *in*-*house* produced biocatalysts were used in the experiments. The enzymes included one endoglucanase of glycoside hydrolase family GH7 (*Tt*EG7) [30], a GH7 cellobiohydrolase (*Tt*CBH7) [13] and an AA9 lytic polysaccharide monooxygenase (*Tt*LPMO) [31]. The enzymes, primarily encoded by the thermophilic fungus *Thermothelomyces thermophila* (previously known as *Myceliophthora thermophila*), were heterologously produced in *Pichia pastoris* as previously described [13, 30, 31].

**Table 10:** Summary of processive enzymes used in the present study

Number	Enzyme name	Short name	Organism	Architecture	Temperature
1	Cellobiohydrolase 48A	*Cc*Cbh48A	*Clostridium cellulolyticum*	GH48	37
2	Cellobiohydrolase 5A	*Ct*Cbh5A	*Clostridium thermocellum*	CBM3-GH5	60
3	Cellulase 9A	*Ct*Cel9A	*Clostridium thermocellum*	GH9	60
4	Cellobiohydrolase 9A	*Ct*Cbh9A	*Clostridium thermocellum*	GH9	60
5	Cellulase 9B	*Ct*Cel9B	*Clostridium thermocellum*	GH9	60
6	Cellobiohydrolase 6A	*Pa*Cbh6A	*Podospora anserina*	CBM1-GH6	50
7	Cellulase 9W	*Cc*Cel9W	*Clostridium cellulolyticum*	GH9	37
8	Cellulase 9 M	*Cc*Cel9M	*Clostridium cellulolyticum*	GH9	37
9	Cellulase 9R	*Cc*Cel9R	*Clostridium cellulolyticum*	GH9	37
10	Cellulase 9A	*Cc*Cel9A	*Clostridium cellulovorans*	GH9	37
11	Cellobiohydrolase 48A	*Cs*Cbh48A	*Clostridium stercorarium*	GH48	60
12	Cellulase 9 J	*Cc*Cel9J	*Clostridium cellulolyticum*	GH9	37
13	Cellulase 9Q	*Cc*Cel9Q	*Clostridium cellulolyticum*	GH9	37
14	Cellulase 9A	*Rf*Cel9A	*Ruminococcus champanellensis*	GH9	50
15	Endo-1,4-β-d-glucanase	EG5	*Talaromyces emersonii*	GH5	50

Organosolv-pretreated birch (**B1**: 200 °C for 15 min, 60% (v/v) EtOH, and **B2**: 200 °C for 30 min, 60% (v/v) EtOH) and spruce (**S1**: 200 °C for 30 min, 52% (v/v) EtOH) were used as substrates [32, 33]. The compositional analysis of the materials was 66.3% (w/w) cellulose, 22% (w/w) hemicellulose, 7.8% (w/w) lignin for **B1**, 67.1% (w/w) cellulose, 21% (w/w) hemicellulose, 7.1% (w/w) lignin for **B2** and 66% (w/w) cellulose, 6% (w/w) hemicellulose, 14.9% (w/w) lignin for **S1** [32, 33]. Carboxymethyl cellulose (CMC), microcrystalline cellulose Avicel PH-101 and cello-oligosaccharides (DP2–6) were obtained from Sigma-Aldrich (USA). Celloheptaose (DP7) and cello-octaose (DP8) were obtained from Elicityl—Oligotech^®^ (France). Phosphoric acid swollen cellulose (PASC) was prepared from Avicel, as previously described [34].

### Screening tests on processive enzymes

In order to select the most promising candidate to be subsequently included in the construction of an enzyme cocktail for the production of cellobiose and other prebiotic cello-oligosaccharides from biomass, initial screening of all processive enzymes against oligosaccharides and polysaccharides was performed. The enzymes were also tested as monoenzymes for their activity on pretreated natural substrates (birch and spruce as forest residues).

#### i. Hydrolysis of polysaccharidic substrates

The activity of all processive enzymes on PASC, Avicel and CMC was evaluated in safe lock microtubes at 1.5 mL reaction volume. Substrates were prepared in 50 mM sodium acetate buffer pH 6.0. The initial substrate concentration was 0.5% (w/v), and the enzyme loading was 20 mg/g substrate. All reactions were performed in 50 mM sodium acetate buffer, under 1100 rpm agitation and contained 0.02% (w/v) NaN_3_. The temperature was set at 37, 50 or 60 °C, according to the enzyme optimal temperature of activity (Table 11). After 24 h, samples were taken, boiled for 5 min for enzyme inactivation, and centrifuged and the supernatant was filtered (0.22 μm pore size). The presence of oligosaccharides was verified with high-performance anion exchange chromatography equipped with pulsed amperometric detection (HPAEC-PAD), as previously described [31]. Evaluation of results was based on the chromatograph peaks corresponding to sugars with DP 1–5.

**Table 11:** Nanofiltration membranes and their characteristics

Manufacturer	Type	Pore size/MWCO^a^ (Da)	Polymer	pH	Flux (GFD/psi)
Dow Filmtec™	NF270	~ 200–400	Polyamide-TFC	2–11	72–98/130
GE Osmonics™	DL	~ 150–300	Polyamide-TFC	2–10	28/220
Synder™	NFX	~ 150–300	Polyamide-TFC	3–10.5	20–25/110
Synder™	NFW	~ 300–500	Polyamide-TFC	4–10	45–50/110
TriSep™	TS40	~ 200	Polypiperazine-amide-TFC	2–11	20/110

^*a*^*MWCO* molecular weight cut-off

The product profile as well as the total cellulose conversion into oligosaccharides was calculated with the following equation (Eq. 1):

1 $${\text{Cellulose}}\;{\text{conversion}} \left( \% \right) = \frac{{\left( {C1 + C2*1.05 + C3*1.07 + C4 *1.08 + C5 *1.09} \right)*100}}{{{\text{Csubstrate}}*1.1}},$$

where the concentration of glucose, oligosaccharides and initial substrate are calculated in mg/mL of reaction volume and 1.05, 1.07, 1.08 and 1.09 are the conversion rates of cellobiose, *C*3, *C*4, and *C*5 to glucose, respectively. The cellulose percentage for PASC, Avicel and CMC was considered as 100%. The degree of processivity (*P*) was calculated by using the equation *P* = (*C*2 − *C*1)/(*C*3 + *C*1), as previously described [18].

#### ii Kinetics of oligosaccharide hydrolysis

The mode of action of the enzymes was evaluated on cello-oligosaccharides with DP 5–8. The initial substrate concentration was 60 mM, and 4 μg of each enzyme was used at a final volume of 1.5 mL. All reactions were performed in 50 mM sodium acetate buffer, contained 0.02% (w/v) NaN_3_. The temperature was set at 37, 50 or 60 °C, according to the enzyme optimal temperature of activity. For the determination of the hydrolysis kinetics, samples were taken every 20 min for a duration of 100 min, boiled for 5 min and filtered and the released oligosaccharides were evaluated with HPAEC/PAD chromatography, as described above. The enzymatic hydrolysis of cello-oligosaccharides was considered as a first-order reaction [35], and the catalytic efficiency of the enzymes against cello-oligosaccharides was estimated by the equation:

2 $$k*t = \ln \frac{{\left[ {\text{So}} \right]}}{{\left[ {\text{St}} \right]}},$$

where *k* = (*k*_cat_/*K*_m_) * [*E*] and [*E*], [So], [St] are calculated in mM and represent the enzyme and the substrate concentration at the beginning of the reaction and at a specified time, respectively [30].

#### iii Activity on lignocellulosic materials

Organosolv-pretreated birch (**B1**, **B2**) and spruce (**S1**) were used as substrates to estimate the activity of the different processive enzymes on lignocellulosic feedstocks. The initial dry matter (DM) was 1.5% (w/v), and the enzyme loading was 8 mg/g substrate. The parameters of the reaction (final volume, buffer, temperature and agitation) were similar to those described above for the hydrolysis of polysaccharidic substrates. Samples were taken at 48 h, boiled, centrifuged and filtered, while the released oligosaccharides were evaluated with HPAEC-PAD chromatography, after the procedure that was previously described [31]. The degree of processivity (*P*) was calculated by using the equation *P* = (*C*2 − *C*1)/(*C*3 + *C*1), as previously described [18].

### Experimental design and cocktail optimization

After selection of the most appropriate processive enzyme to be included in the subsequent design, the cello-oligosaccharide yields from organosolv-pretreated biomass using different enzyme mixtures were studied. Preliminary tests were conducted in order to set the upper and lower concentrations for each enzyme. Hydrolysis of birch and spruce (B1, B2 and S1) was performed using different combinations of two *in*-*house* produced enzymes (*Tt*LPMO, *Tt*CBH7) and the commercially available endoglucanase EG5. Enzymatic reactions were performed in safe lock microtubes at 1.5 mL reaction volume, at 50 °C, under agitation of 1100 rpm, with 3% (w/v) initial DM. The enzymes were loaded at 25 mg/g substrate. All reactions were performed in 50 mM sodium acetate buffer pH 6.0 and contained 0.02% (w/v) NaN_3_. Samples were taken at 24 h and further processed as described above. As the main reaction products were cellobiose and glucose, sugar analysis was performed by isocratic ion-exchange chromatography, using an Aminex HPX-87H column with a micro-guard column, at 65 °C (Bio-Rad Laboratories, Hercules, CA, USA), as previously described [36]. Cellulose conversion into cellobiose was calculated by following the equation below:

3 $${\text{Cellulose}} \;{\text{conversion}} \, \left( \% \right) = \frac{C2*1.05*100}{{C_{\text{substrate}} *\% \, {\text{cellulose}}*1.1}} ,$$

where the concentration of cellobiose and initial substrate is calculated in mg/mL of reaction volume and 1.05 is the conversion rates of cellobiose to glucose. The percentage of cellulose for each substrate is described above, in Sect. "Enzymes and substrates".

For this experimental design and the cocktail optimization, four enzymes, including the most promising processive enzyme candidate that was chosen after the screening studies, EG5, *Tt*CBH7, and *Tt*LPMO were used. The upper and lower limits of the percentage of each enzyme were defined based on data from previous studies [13, 37] (Additional file 1: Table S6), and preliminary results conducted with EG5, *Tt*CBH7 and *Tt*LPMO, as described above. The different enzyme combinations are described in Additional file 1: Table S7. The *in*-*house* produced endoglucanase *Mt*EG7 was also used in all enzyme combinations, at an amount equal to 5% (w/w) of the total cocktail. The reactions were performed with 3% (w/v) initial dry matter and a total enzyme loading of 25 mg/g substrate in 50 mM sodium acetate buffer pH 6.0, as described above. Samples were taken at 24 and 48 h. After centrifugation and boiling, the supernatants were filtered (0.22 μm pore size) and the released sugars were detected by HPLC chromatography using an Aminex HPX-87H column as described above. The evaluation of the results toward the maximum cellobiose yields was performed with Design Expert 7.0.0 software using the most appropriate model that fits the experimental data [13, 37]. *D*-optimal design was used to fit the results. The predicted enzyme combinations that could yield the optimal cellobiose production were verified experimentally.

### Nanofiltration tests

A nanofiltration system comprised of a HP4750 high-pressure stirred cell and various membranes (Sterlitech, USA) was employed. The nanofiltration vessel was a magnetically stirred dead-end stainless-steel cell with a working volume of 300 mL and effective membrane area of 14.6 cm^2^. A constant pressure of 5, 10, 15 or 20 bar was provided by filling nitrogen gas into the cell, while the permeate was collected in a beaker placed on an electronic scale in order to calculate the permeate flux. In the case of higher temperature conditions (40, 50 or 60 °C), the nanofiltration vessel was placed in a water bath. The membranes that were used in this study, as well as their characteristics, are described in Table 11. For testing the membranes, d-glucose (180.156 g/mol) and d-cellobiose (342.3 g/mol) were purchased from Sigma-Aldrich (USA) and were used for the model solutions.

#### i. Water permeability measurements

For the evaluation of water permeability properties, the membranes, stored in 1% (w/v) sodium metabisulfite, were rinsed with dH_2_O and placed in the nanofiltration dead-end cell. Then, the cell was filled with 50 mL dH_2_O and a pressure of 10 bar was applied. Measurements of the permeate’s weight were taken every 5 min for 20 min in total. The water permeability of each membrane was determined every time before and after the use of the membrane as an indication of the membrane efficiency; whenever a change was detected the membrane was discarded and replaced by a new one. The tests for the water permeability were conducted at room temperature.

The permeate’s flux was calculated according to the equation (Eq. 4):

4 $$J = \frac{V}{A*t} ,$$

where *J* represents the permeate flux (L/m^2^ h), V the permeate volume (L), A the membrane area (m^2^), and t the filtration time (h) [38].

The water permeability of the membrane (*L*_p_) was calculated by the equation (Eq. 5):

5 $$L_{\text{p}} = \frac{{J_{\text{w}} }}{\text{TMP}} ,$$

where *J*_w_ represents the water flux (L/h) and TMP the transmembrane pressure (m^2^ * bar).

#### Nanofiltration of model solutions and hydrolysis products

The model solutions that were used for the experiments were prepared with commercial glucose and cellobiose dissolved in dH_2_O. The feed concentration that was tested was 5 and 20 mg/mL. The filtration was performed at concentration ratio of cellobiose to glucose 9:1, at different pressure conditions (5, 10, 15 and 20 bar), and the experiments were carried out at room temperature. The membranes that were more suitable for the experimental purposes and led to efficient separation of cellobiose and glucose were further tested at different temperatures (40, 50 and 60 °C). The feed volume was 50 mL. During the nanofiltration process, the feed solutions were concentrated by a factor of 2 and samples of the permeate were taken every 15 min on average depending on the flow rate of the permeate. At the end of the filtration, another sample was taken from the sugar mixture that was inside of the vessel (retentate). All samples were then filtrated with 0.22-μm-pore-size filters and were analyzed with HPLC chromatography using an Aminex HPX-87H column, as previously described [36].

The retention of a solute *j* (*R*_*j*_) is obtained by the following equation:

6 $$R_{j} = \left( {1 - \frac{{C_{{{\text{p}},j}} *V_{\text{p}} }}{{C_{{{\text{F}},j}} *V_{\text{F}} }}} \right)*100\% ,$$

whereas *C*_p,*j*_ is the concentration of the solute *j* in the permeate and *C*_F,*j*_ the concentration of the solute *j* in the feed. *V*_p_ is the volume of the permeate, and *V*_F_ is the volume of the feed. For the calculations, the average original feed and final retentate concentration were used, as the calculation based on the time-dependent mass balance during the trials was not easy to be determined.

The cellobiose separation factor is given by the following equation, where the observed percentage retention values of cellobiose (*R*_cell_) and glucose (*R*_gl_) are used. A separation factor greater than one corresponds to cellobiose enrichment in the retentate as compared to the feed solution [24]:

7 $$X_{\text{cell}} = \frac{{R_{\text{cell}} }}{{R_{\text{gl}} }} .$$

Another screening of the membranes was performed using as feed a “real” lignocellulose-derived stream, more specifically a birch hydrolysate after enzymatic treatment with a commercially available cellulase mixture from *Trichoderma reesei* (Celluclast^®^, Sigma-Aldrich). The aim of this test was to evaluate the performance of the membranes toward the separation of cellobiose and glucose under conditions that could mimic the real condition and, moreover, to compare the results with those in the case of the pure sugars model solution. The hydrolysis solution was produced by a 50 mL reaction with 3% (w/v) initial DM, 25 mg/g substrate enzyme loading and 50 mM sodium acetate pH 5.0 after 48 h of incubation. Then, it was boiled, centrifuged, filtrated with 0.22-μm-pore-size filter and was then used for the nanofiltration trials. The trials were carried out at the optimal pressure condition of 10 bar and at room temperature for all the membranes except for the membrane NFW which showed very low performance on the previous trials with the model solution. The membranes with high performance and separation efficiency were further studied at higher temperatures (40, 50 and 60 °C). Nanofiltration trials were conducted as described above, and samples from the retentate and the permeate were analyzed by HPLC chromatography.

### Scale-up reaction with 6% (w/v) DM and product recovery

After identifying the optimal enzyme combination that could maximize the cellobiose yield on birch and spruce biomass, a scale-up reaction was set up by employing the optimal enzyme ternary mixture. The initial dry matter was 6% (w/v) and the enzyme loading was 25 mg/g substrate, all suspended in 15 mM ammonium acetate buffer pH 6.0. The reaction total volume was 100 mL, and the ratio of reaction volume to shake flask volume was 1/10. The hydrolysis took place at 50 °C, under continuous agitation of 170 rpm. The enzymes were loaded in two steps sequentially in order to maximize hydrolysis and prevent end-product inhibition, by maintaining the same total enzyme loading. First, an enzyme loading of 15 mg/g was added and was incubated for 48 h. Then, after centrifugation, the hydrolysate was collected, the residual biomass was washed with the same buffer, and the washed hydrolysate was also collected. There was no boiling step at this stage, as the enzymes were collected through ultrafiltration (see below). The washed biomass was again placed in the shake flask, and the residual 10 mg/g enzyme loading was added together with the proper amount of buffer for another 48 h. Then, the same procedure for washing the biomass was followed. All the hydrolysates (initial and after wash) were filtrated with 0.22-μm-pore-size filter, and then, samples were taken for HPLC analysis for identifying and quantifying the cellobiose and glucose content using an Aminex HPX-87H column, as previously described [19]. Then, all were mixed in one hydrolysate that was further processed to ultrafiltration for the removal of the enzymes and nanofiltration for the glucose removal.

For the removal of the total protein, the hydrolysate was filtrated with a LabScale Tangential Flow Filtration system (TFF) (Millipore) with exclusion membrane size 5 kDa (Pellicon XL Ultrafiltration Module Biomax 5 kDa, Millipore). The retentate, containing the concentrated solution of cellulases, was maintained in 4 °C for further use in other hydrolysis experiments. The permeate was then collected and applied to nanofiltration system by using the NF270 membrane. The retentate was collected, freeze-dried and stored in a dry place until further use. Samples from all the different steps of the process were collected and analyzed by HPLC [36] in order to determine the sugar content and the presence of acids originating from biomass components (hemicellulose) or reaction conditions (buffer).

### Evaluation of prebiotic activity of COS

Birch- and spruce-derived cello-oligosaccharides (COS) produced after enzymatic hydrolysis were used for prebiotic tests at an initial concentration of 2% (w/v). The hydrolysis products were tested whether they could be utilized as carbon sources and support the growth of probiotic strains. Six bacterial strains belonging to the *lactobacilli* and *bifidobacteria* species (3 strains for each species, respectively) were included in this study as they are representative species with probiotic properties. *Bifidobacterium adolescentis* DSM 20083, *Bifidobacterium longum* DSM 20219, *Lactobacillus gasseri* DSM 20077 and *Lactobacillus reuteri* DSM 20016 were purchased from DSMZ (Braunschweig, Germany). *Bifidobacterium animalis* subsp. lactis was a generous offer from Essum Probiotics AB, Umeå, Sweden. *Lactobacillus plantarum* ATCC 8014 was obtained from ATCC (VA, USA). The growth medium for the *Lactobacillus* strains stock cultures was MRS medium with cysteine (Medium 232 DSMZ), while in the case of *Bifidobacteria* strains, the *Bifidobacterium* medium (Medium 58 DSMZ) was used. When cellobiose and COS were tested as carbon source, both bacterial species were cultivated in *in*-*house* prepared MRS broth in the absence of glucose or any other carbohydrate. The pH of the medium was adjusted to 6.0.

Cultivation took place anaerobically at the optimal growth temperature of 37 °C from stock cultures. The stock cultures were maintained at − 80 °C in the appropriate medium supplemented with 50% (v/v) glycerol as a cryoprotectant. Cells from the culture broth of each species were subcultured (5% v/v) into fresh media and incubated 24 h anaerobically at 37 °C. Growth rate was monitored by identifying the optical density of 600 nm (OD_600_). The OD of the preculture was measured, and the volume of the inoculum was then calculated in order for the starting OD of the main culture to be equal to 0.3. To test the effectiveness of cellobiose as prebiotic candidate, the growth of all strains on pure cellobiose as carbon source was tested as a positive control. The cells from the glucose precultures were collected after centrifugation at 4000 rpm for 10 min and resuspended in 50 mL MRS medium containing 2% (w/v) cellobiose instead of glucose. The incubation for all the cultures was achieved in a vinyl anaerobic chamber (COY Laboratory products, USA) under strict anaerobic atmosphere using a regulated gas mixture, for a maximum of 190 h. Growth rate was monitored by identifying the OD_600_, while sugar consumption and release of fermentation products [lactic acid and other short chain fatty acids (SCFA)] were analyzed using HPLC chromatography with Aminex HPX-87H column as described above [36]. All trials were run in duplicates. Cultures with MRS media with 2% (w/v) glucose, as well as in the absence of any carbon source, were used for comparison.

The selected strains (*Lactobacilli* strains) that were able to efficiently grow on cellobiose were further tested on birch- and spruce-derived COS. 2% (w/v) cellobiose content media were prepared from the final hydrolysis products of the scale-up reactions, where the sugars were dissolved directly in MRS broth. The final product from spruce hydrolysis reaction was proved to be difficulty dissolved and required heating to 60 °C. The obtained media were then sterilized by membrane filtration using 0.22-μm-pore-size filters. The reaction conditions were similar to those followed for pure cellobiose, while the determination of growth rate and production of metabolites were conducted as described above.

## Supplementary information

**Additional file 1: Table S1.** Product profile and % w/w cellulose conversion into soluble oligosaccharides from pure cellulosic substrates. **Table S2.** Product profile and % w/w cellulose conversion into soluble oligosaccharides from lignocellulosic substrates. **Table S3.** Cellobiose yield for 24 and 48 h of hydrolysis of organosolv-pretreated spruce (S1) and birch (B1). The response factor (% w/w cellobiose) refers to the % w/w conversion of cellulosic biomass content into cellobiose. **Table S4.** Final equations in terms of actual components for spruce (S1) and birch (B1) experimental design. **Table S5.** Average water permeability for all the membranes. **Table S6.** Upper and lower limits for all variables used for the experimental design. **Table S7.** Enzyme combinations for the optimization tests generated with D-optimal experimental design (Design Expert® 7.0.0, Stat-Ease Inc.). **Figure S1.** Ternary plots displaying the predicted cellobiose concentration (mg/mL) from birch hydrolysis at 24 (A) and 48 h (B), as a function of three out of four enzymes. For each plot, the fourth enzyme (“Actual Component”) is set at the relative proportion of the point yielding the maximum amount of cellobiose, as predicted by the statistical model. **Figure S2.** Ternary plots displaying the predicted cellobiose concentration (mg/mL) from spruce hydrolysis at 24 (A) and 48 h (B), as a function of three out of four enzymes. For each plot, the fourth enzyme (“Actual Component”) is set at the relative proportion of the point yielding the maximum amount of cellobiose, as predicted by the statistical model.

## Data Availability

COS from enzymatic hydrolysis of birch biomass are available upon reasonable request.

## Abbreviations

COS: cello-oligosaccharides
EG: endoglucanase
CBH: cellobiohydrolase
LPMO: lytic polysaccharide monooxygenase
PASC: phosphoric-acid swollen cellulose
CMC: carboxymethyl cellulose
