# Supplementary material for: Valorization of waste forest biomass toward the production of cello-oligosaccharides with potential prebiotic activity by utilizing customized enzyme cocktails
Source: Biotechnol Biofuels. 2019 Dec 10;12:285. doi: 10.1186/s13068-019-1628-z (PMC6902470; doi:10.1186/s13068-019-1628-z)
Supplement: Supplementary file 1 — Additional file 1: Table S1. Product profile and % w/w cellulose conversion into soluble oligosaccharides from pure cellulosic substrates. Table S2. Product profile and % w/w cellulose conversion into soluble oligosaccharides from lignocellulosic substrates. Table S3. Cellobiose yield for 24 and 48 h of hydrolysis of organosolv-pretreated spruce (S1) and birch (B1). The response factor (% w/w cellobiose) refers to the % w/w conversion of cellulosic biomass content into cellobiose. Table S4. Final equations in terms of actual components for spruce (S1) and birch (B1) experimental design. Table S5. Average water permeability for all the membranes. Table S6. Upper and lower limits for all variables used for the experimental design. Table S7. Enzyme combinations for the optimization tests generated with D-optimal experimental design (Design Expert® 7.0.0, Stat-Ease Inc.). Figure S1. Ternary plots displaying the predicted cellobiose concentration (mg/mL) from birch hydrolysis at 24 (A) and 48 h (B), as a function of three out of four enzymes. For each plot, the fourth enzyme (“Actual Component”) is set at the relative proportion of the point yielding the maximum amount of cellobiose, as predicted by the statistical model. Figure S2. Ternary plots displaying the predicted cellobiose concentration (mg/mL) from spruce hydrolysis at 24 (A) and 48 h (B), as a function of three out of four enzymes. For each plot, the fourth enzyme (“Actual Component”) is set at the relative proportion of the point yielding the maximum amount of cellobiose, as predicted by the statistical model. [file 13068_2019_1628_MOESM1_ESM.docx]

Valorization of waste forest biomass towards the production of cello-oligosaccharides with prebiotic potential by utilizing customized enzyme cocktails

Anthi Karnaouri, Leonidas Matsakas, Eleni Krikigianni, Ulrika Rova and Paul Christakopoulos

**Additional file 1**

**Table S1**. Product profile and % w/w cellulose conversion into soluble oligosaccharides from pure cellulosic substrates.

|  | **CMC** | | | | | | | | **PASC** | | | | | | |
| --- | --- | --- | --- | --- | --- | --- | --- | --- | --- | --- | --- | --- | --- | --- | --- |
|  |  | **Products (%)** | | | | | | **% conversion** | **Products (%)** | | | | | **% converson** | |
| **Enzyme name** | | **C1** | **C2** | | **C3** | **C4** | **C5** |  | **C1** | **C2** | **C3** | **C4** | **C5** |  |  |
| 1 | *CcCel48A* | 0 | | 49.4 | 25.7 | 24.9 | 0 | 0.22 | 1 | 57.2 | 21.5 | 20.4 | 0 | 2.57 |  |
| 2 | *CtCbh5A* | 3.4 | | 78.3 | 12.4 | 5.9 | 0 | 1.61 | 2.1 | 71.1 | 26.2 | 0.7 | 0 | 5.93 |  |
| 3 | *CtCel9A* | 0 | | 33.7 | 18.5 | 31.5 | 16.3 | 0.55 | 11.7 | 47.7 | 14.2 | 26.4 | 0 | 0.16 |  |
| 4 | *CtCbh9A* | 8.4 | | 44.8 | 36.8 | 7.6 | 2.5 | 0.38 | 0 | 44.2 | 55.8 | 0 | 0 | 0.03 |  |
| 5 | *CtCel9B* | 0 | | 70.8 | 27.4 | 1.8 | 0 | 1.01 | 0 | 75.4 | 19.8 | 4.8 | 0 | 0.88 |  |
| 6 | *PaCbh6A* | 2.5 | | 85.9 | 13.6 | 0 | 0 | 0.62 | 2.2 | 87.2 | 10.4 | 0 | 0 | 6.7 |  |
| 7 | *CcCel9W* | 7.7 | | 53.6 | 34.5 | 4.2 | 0 | 1.01 | 5.9 | 40.2 | 36.1 | 13 | 4.9 | 3.4 |  |
| 8 | *CcCel9M* | 5.8 | | 25.2 | 18.9 | 24.7 | 25.4 | 1.45 | 11.4 | 30 | 23.6 | 35 | 0 | 6.77 |  |
| 9 | *CcCel9R* | 1.3 | | 14.8 | 28.9 | 35.8 | 19.2 | 0.93 | 1 | 16 | 25.4 | 41.2 | 16.4 | 1.49 |  |
| 10 | *CcCel9A* | 11.2 | | 81.4 | 7.4 | 0 | 0 | 0.98 | 14.4 | 80.7 | 3.7 | 1.2 | 0 | 5.13 |  |
| 11 | *CsCbh48A* | 5.2 | | 59.5 | 21 | 5.9 | 8.4 | 1 | 2.1 | 70.2 | 19.7 | 6.1 | 1.8 | 3.34 |  |
| 12 | *CcCel9J* | 0 | | 0 | 0 | 0 | 0 | 0 | 0 | 0 | 0 | 0 | 0 | 0 |  |
| 13 | *CcCel9Q* | 6.7 | | 59 | 22.8 | 0 | 11.6 | 1.02 | 6.7 | 60.1 | 26.8 | 3.4 | 3 | 3.79 |  |
| 14 | *RfCel9A* | 1.8 | | 44.2 | 20.1 | 24.9 | 9 | 1.34 | 1.9 | 35.5 | 19.1 | 32 | 11.5 | 2.47 |  |

|  | **Avicel** | | | | | | |
| --- | --- | --- | --- | --- | --- | --- | --- |
|  |  | **Products (%)** | | | | | **% conversion** |
| **Enzyme name** | | **C1** | **C2** | **C3** | **C4** | **C5** |  |
| 1 | *CcCel48A* | 1.3 | 61.6 | 29.8 | 7.3 | 0 | 0.37 |
| 2 | *CtCbh5A* | 4.1 | 79 | 16.8 | 0 | 0 | 0.44 |
| 3 | *CtCel9A* | 0 | 52.2 | 24.1 | 23.6 | 0 | 0.06 |
| 4 | *CtCbh9A* | 0 | 0 | 100 | 0 | 0 | 0.02 |
| 5 | *CtCel9B* | 0 | 70.1 | 29.9 | 0 | 0 | 0.11 |
| 6 | *PaCbh6A* | 2.5 | 92.5 | 5 | 0 | 0 | 1.1 |
| 7 | *CcCel9W* | 7.6 | 50.7 | 41.6 | 0 | 0 | 0.24 |
| 8 | *CcCel9M* | 12.5 | 36 | 50.1 | 1.4 | 0 | 0.75 |
| 9 | *CcCel9R* | 5.2 | 30 | 42.6 | 22.3 | 0 | 0.05 |
| 10 | *CcCel9A* | 3.5 | 85.2 | 11.3 | 0 | 0 | 0.45 |
| 11 | *CsCbh48A* | 1.8 | 75.8 | 22.5 | 0 | 0 | 0.63 |
| 12 | *CcCel9J* | 0 | 0 | 0 | 0 | 0 | 0 |
| 13 | *CcCel9Q* | 1.4 | 74 | 24.7 | 0 | 0 | 0.21 |
| 14 | *RfCel9A* | 2.2 | 56.9 | 18.4 | 22.5 | 0 | 0.07 |

**no C5 products were released when Avicel was used as a substrate*

**Table S2**. Product profile and % w/w cellulose conversion into soluble oligosaccharides from lignocellulosic substrates.

|  | **B1*** | | | | | | | | **B2*** | | | | | | |
| --- | --- | --- | --- | --- | --- | --- | --- | --- | --- | --- | --- | --- | --- | --- | --- |
|  |  | **Products (%)** | | | | | | **% conversion** | **Products (%)** | | | | | **% converson** | |
| **Enzyme name** | | **C1** | **C2** | | **C3** | **C4** | **C5** |  | **C1** | **C2** | **C3** | **C4** | **C5** |  |  |
| 1 | *CcCel48A* | 0 | | 90.9 | 9.1 | 0 | 0 | 0.19 | 0 | 85 | 6 | 9.1 | 0 | 0.24 |  |
| 2 | *CtCbh5A* | 16.4 | | 77.6 | 4.2 | 1.7 | 0 | 1.43 | **19.9** | **78.5** | **0.8** | **0.7** | 0 | 3.54 |  |
| 3 | *CtCel9A* | 0 | | 0 | 0 | 0 | 0 | 0 | 0 | 0 | 0 | 0 | 0 | 0 |  |
| 4 | *CtCbh9A* | 0 | | 0 | 100 | 0 | 0 | 0.03 | 0 | 1.5 | 100 | 0 | 0 | 0.06 |  |
| 5 | *CtCel9B* | 0 | | 76.6 | 23.4 | 0 | 0 | 0.04 | 0 | 71.2 | 29.8 | 0 | 0 | 0.04 |  |
| 6 | *PaCbh6A* | 0 | | 94.9 | 5.1 | 0 | 0 | 0.51 | **6.9** | **92.3** | **0.8** | **0** | 0 | 0.89 |  |
| 7 | *CcCel9W* | 0 | | 67.2 | 16.6 | 16.2 | 0 | 0.78 | 10.3 | 79.6 | 1 | 9 | 0 | 1.35 |  |
| 8 | *CcCel9M* | 33.5 | | 41.1 | 23.8 | 1.7 | 0 | 0.99 | 34.6 | 61.4 | 1 | 3.1 | 0 | 2.02 |  |
| 9 | *CcCel9R* | 89.5 | | 0 | 4.9 | 5.6 | 0 | 0.16 | 89.2 | 0 | 0.7 | 10.1 | 0 | 0.11 |  |
| 10 | *CcCel9A* | 10.6 | | 80.2 | 5.6 | 3.5 | 0 | 1.3 | 13.2 | 84.6 | 0.8 | 1.3 | 0 | 1.85 |  |
| 11 | *CsCbh48A* | 2.5 | | 87.4 | 10.1 | 0 | 0 | 0.47 | 8.9 | 87.8 | 0.8 | 2.4 | 0 | 0.56 |  |
| 12 | *CcCel9J* | 0 | | 0 | 0 | 0 | 0 | 0 | 0 | 0 | 0 | 0 | 0 | 0 |  |
| 13 | *CcCel9Q* | 2.3 | | 63.8 | 16.4 | 17.5 | 0 | 0.81 | 13.6 | 73.8 | 0.9 | 11.7 | 0 | 0.76 |  |
| 14 | *RfCel9A* | 0 | | 0 | 0 | 0 | 0 | 0 | 0 | 0 | 0 | 0 | 0 | 0 |  |

**no C5 products were released*

|  | **S1*** | | | | | | |
| --- | --- | --- | --- | --- | --- | --- | --- |
|  |  | **Products (%)** | | | | | **% conversion** |
| **Enzyme name** | | **C1** | **C2** | **C3** | **C4** | **C5** |  |
| 1 | *CcCel48A* | 0 | 73.6 | 15.6 | 10.7 | 0 | 0.9 |
| 2 | *CtCbh5A* | **16.2** | **81** | **2.8** | **0** | 0 | 3.18 |
| 3 | *CtCel9A* | 0 | 58 | 31 | 11 | 0 | 0.05 |
| 4 | *CtCbh9A* | 0 | 0 | 100 | 0 | 0 | 0.02 |
| 5 | *CtCel9B* | 0 | 59.6 | 29.1 | 11.3 | 0 | 0.12 |
| 6 | *PaCbh6A* | **0** | **92.1** | **7.9** | **0** | 0 | 1.86 |
| 7 | *CcCel9W* | 0 | 63.4 | 27.7 | 8.9 | 0 | 0.63 |
| 8 | *CcCel9M* | 33.3 | 42.2 | 22.9 | 1.5 | 0 | 1.94 |
| 9 | *CcCel9R* | 0 | 26.6 | 25.6 | 47.8 | 0 | 0.13 |
| 10 | *CcCel9A* | 10.7 | 86.8 | 2.5 | 0 | 0 | 2.06 |
| 11 | *CsCbh48A* | 5 | 86.2 | 8.7 | 0 | 0 | 1.68 |
| 12 | *CcCel9J* | 0 | 0 | 0 | 0 | 0 | 0 |
| 13 | *CcCel9Q* | 12.2 | 77.2 | 10.6 | 0 | 0 | 0.85 |
| 14 | *RfCel9A* | 0 | 53.9 | 15.5 | 30.6 | 0 | 0.15 |

**no C5 products were released*

**Table S3**. Cellobiose yield for 24 and 48 h of hydrolysis of organosolv pretreated spruce (S1) and birch (B1). The response factor (% w/w cellobiose) refers to the % w/w conversion of cellulosic biomass content into cellobiose.

|  |  |  |  |  | **Spruce (S1)** | | **Birch (B1)** | |
| --- | --- | --- | --- | --- | --- | --- | --- | --- |
|  |  |  |  |  | % cellobiose conversion | | % cellobiose conversion | |
|  |  |  |  |  | 24h | 48h | 24h | 48h |
| **# run** | **EG5** | ***Mt*EG7** | ***Tt*LPMO** | ***Pa*Cbh6A** | **cellobiose** | **cellobiose** | **cellobiose** | **cellobiose** |
| 1 | 0.2 | 0.6 | 0.1 | 0.1 | 11.87 | 15.42 | 16.55 | 21.62 |
| 2 | 0.2 | 0.6 | 0.1 | 0.1 | 12.13 | 16.47 | 15.02 | 21.32 |
| 3 | 0.3 | 0.3 | 0.3 | 0.1 | 11.7 | 15.23 | 14.73 | 18.74 |
| 4 | 0.5 | 0.3 | 0.1 | 0.1 | 12.18 | 16.52 | 15.73 | 20.57 |
| 5 | 0.3 | 0.3 | 0.2 | 0.2 | 12.07 | 17.55 | 15.66 | 19.57 |
| 6 | 0.4 | 0.3 | 0.2 | 0.1 | 11.89 | 16.25 | 12.91 | 17.02 |
| 7 | 0.2 | 0.3 | 0.3 | 0.2 | 11.47 | 14.77 | 10.03 | 13.84 |
| 8 | 0.2 | 0.3 | 0.3 | 0.2 | 11.16 | 14.72 | 10.61 | 15.32 |
| 9 | 0.2 | 0.5 | 0.1 | 0.2 | 11.35 | 16.17 | 14.04 | 18.09 |
| 10 | 0.5 | 0.3 | 0.1 | 0.1 | 12.22 | 15.46 | 14.43 | 18.91 |
| 11 | 0.2 | 0.4 | 0.3 | 0.1 | 10.97 | 20.49 | 15.46 | 20.44 |
| 12 | 0.2 | 0.4 | 0.2 | 0.2 | 11.13 | 18.27 | 15.21 | 19.54 |
| 13 | 0.35 | 0.45 | 0.1 | 0.1 | 12.08 | 20.01 | 13.8 | 18.35 |
| 14 | 0.2 | 0.4 | 0.1 | 0.3 | 10.72 | 18.31 | 12.17 | 17.1 |
| 15 | 0.3 | 0.3 | 0.1 | 0.3 | 11.07 | 17.72 | 15.7 | 20.62 |
| 16 | 0.35 | 0.45 | 0.1 | 0.1 | 12.07 | 19.21 | 16.21 | 20.19 |
| 17 | 0.4 | 0.3 | 0.1 | 0.2 | 12.02 | 18.92 | 15.48 | 19.86 |
| 18 | 0.2 | 0.5 | 0.2 | 0.1 | 10.99 | 19.31 | 16.77 | 21.49 |
| 19 | 0.2 | 0.3 | 0.2 | 0.3 | 11.42 | 17.68 | 12.43 | 17.88 |
| 20 | 0.2 | 0.3 | 0.2 | 0.3 | 10.83 | 17.27 | 13.45 | 16.73 |

**Table S4**. Final equations in terms of actual components for spruce (S1) and birch (B1) experimental design

| BIRCH  **24h: Quadratic model**  R^2^ 0.7404, p-value 0.0434  cellobiose =  +6.37643 * EG5  +15.90914 * CBH7  -73.83183 * LPMO  +19.27855 * PaCbh6a  -8.48490 * EG5 * CBH7  +102.75971 * EG5 * LPMO  +87.68125 * EG5 * PaCbh6a  +188.61163 * CBH7 * LPMO  -65.57469 * CBH7 * PaCbh6a  +20.34050 * LPMO * PaCbh6a | **48h: Quadratic model**  R^2^ 0.8071, p-value 0.0124  cellobiose =  +21.85796 * EG5  +31.41960 * CBH7  -51.28530 * LPMO  +54.86007 * PaCbh6a  -46.48201 * EG5 * CBH7  +55.75917 * EG5 * LPMO  +48.84063 * EG5 * PaCbh6a  +161.26014 * CBH7 * LPMO  -132.38860 * CBH7 * PaCbh6a  -51.21379 * LPMO * PaCbh6a |
| --- | --- |

| SPRUCE  **24h: Quadratic model**  R^2^ 0.9072, p-value 0.0004  cellobiose =  +10.01976 * EG5  +15.47736 * CBH7  +10.47938 * LPMO  -4.57792 * PaCbh6a  -0.055315 * EG5 * CBH7  +14.23771 * EG5 * LPMO  +28.09747 * EG5 * PaCbh6a  -24.27796 * CBH7 * LPMO  +4.90633 * CBH7 * PaCbh6a  +41.03493 * LPMO * PaCbh6a | **48h: Quadratic model**  R^2^ 0.8711, p-value 0.0008  cellobiose =  -22.57162 * EG5  -23.41113 * CBH7  -61.74409 * LPMO  +44.75512 * PaCbh6a  +163.20531 * EG5 * CBH7  +52.68479 * EG5 * LPMO  +35.67206 * EG5 * PaCbh6a  +262.32610 * CBH7 * LPMO  -16.42737 * CBH7 * PaCbh6a  -12.09445 * LPMO * PaCbh6a |
| --- | --- |

**Table S5.** Average water permeability for all the membranes. The trials were carried out at 10 bar and room temperature conditions.

| Membrane | Water permeability (L_p_) |
| --- | --- |
| NF270 | 8 |
| DL | 7 |
| NFX | 6 |
| NFW | 13 |
| TS40 | 8 |

**Table S6.** Upper and lower limits for all variables used for the experimental design.

|  | Model variable | Upper limit | Lower limit |  |
| --- | --- | --- | --- | --- |
| EG5 | A | 0.2 | 0.5 |  |
| *Tt*CBH7 | B | 0.3 | 0.6 |  |
| *Tt*LPMO | C | 0.1 | 0.3 |  |
| *Pa*Cbh6a | D | 0.1 | 0.3 |  |
|  |  |  |  | |

**Table S7.** Enzyme combinations for the optimization tests generated with D-optimal experimental design (Design Expert® 7.0.0, Stat-Ease Inc.)

| # run | Enzyme proportions | | | | |
| --- | --- | --- | --- | --- | --- |
|  | EG5 | *TtCBH7* | *Tt*LPMO | *PaCbh6a* | *Mt*EG7 |
| 1 | 0.19 | 0.57 | 0.095 | 0.095 | 0.05 |
| 2 | 0.19 | 0.57 | 0.095 | 0.095 | 0.05 |
| 3 | 0.285 | 0.285 | 0.285 | 0.095 | 0.05 |
| 4 | 0.475 | 0.285 | 0.095 | 0.095 | 0.05 |
| 5 | 0.285 | 0.285 | 0.19 | 0.19 | 0.05 |
| 6 | 0.38 | 0.285 | 0.19 | 0.095 | 0.05 |
| 7 | 0.19 | 0.285 | 0.285 | 0.19 | 0.05 |
| 8 | 0.19 | 0.285 | 0.285 | 0.19 | 0.05 |
| 9 | 0.19 | 0.475 | 0.095 | 0.19 | 0.05 |
| 10 | 0.475 | 0.285 | 0.095 | 0.095 | 0.05 |
| 11 | 0.19 | 0.38 | 0.285 | 0.095 | 0.05 |
| 12 | 0.19 | 0.38 | 0.19 | 0.19 | 0.05 |
| 13 | 0.333 | 0.428 | 0.095 | 0.095 | 0.05 |
| 14 | 0.19 | 0.38 | 0.095 | 0.285 | 0.05 |
| 15 | 0.285 | 0.285 | 0.095 | 0.285 | 0.05 |
| 16 | 0.333 | 0.428 | 0.095 | 0.095 | 0.05 |
| 17 | 0.38 | 0.285 | 0.095 | 0.19 | 0.05 |
| 18 | 0.19 | 0.475 | 0.19 | 0.095 | 0.05 |
| 19 | 0.19 | 0.285 | 0.19 | 0.285 | 0.05 |
| 20 | 0.19 | 0.285 | 0.19 | 0.285 | 0.05 |

**Figure S1**. Ternary plots displaying the predicted cellobiose concentration (mg/mL) from birch hydrolysis at 24 (A) and 48 h (B), as a function of three out of four enzymes. For each plot, the fourth enzyme (‘Actual Component’) is set at the relative proportion of the point yielding the maximum amount of cellobiose, as predicted by the statistical model.

**(A)**


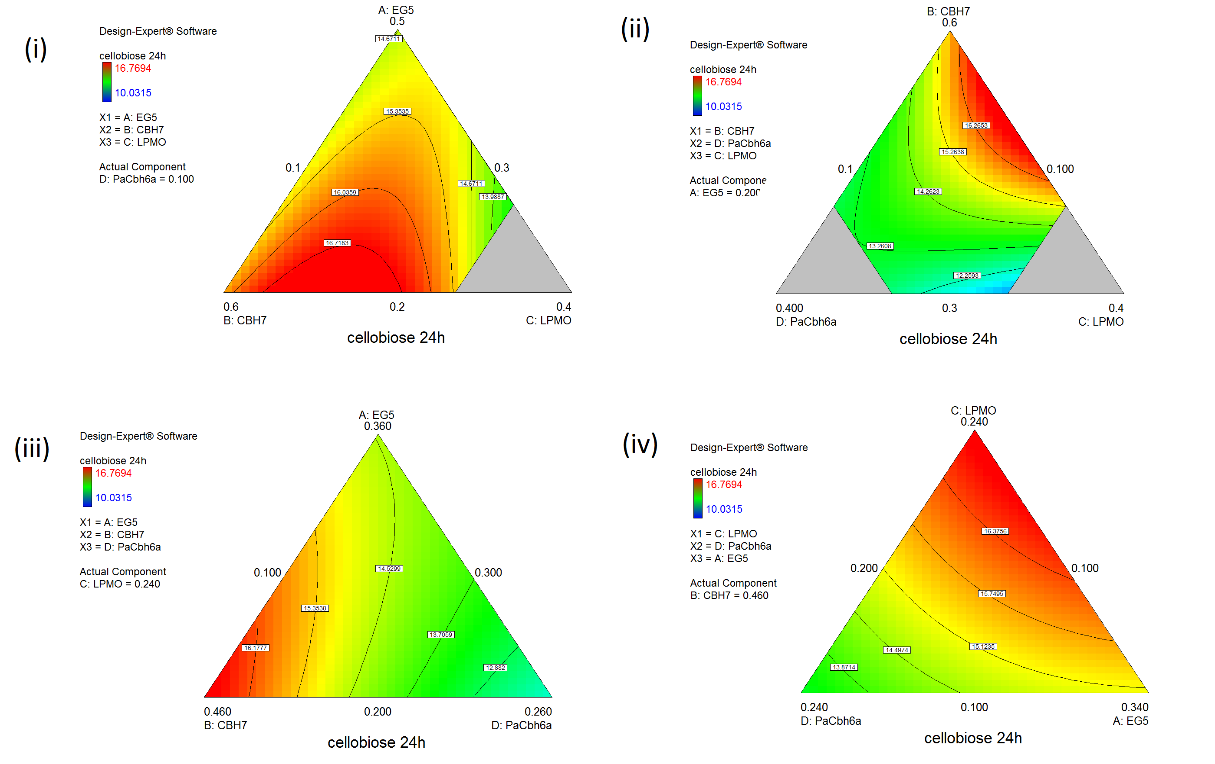


**(B)**


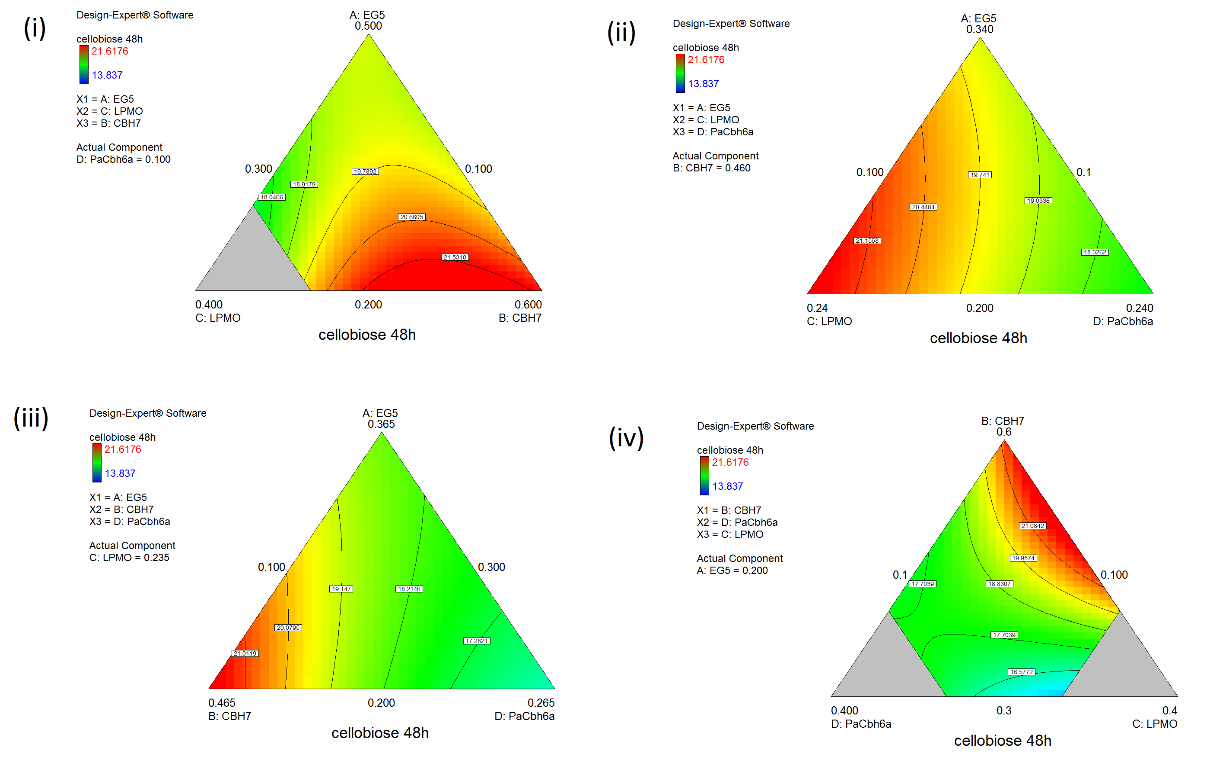


**Figure S2**. Ternary plots displaying the predicted cellobiose concentration (mg/mL) from spruce hydrolysis at 24 (A) and 48 h (B), as a function of three out of four enzymes. For each plot, the fourth enzyme (‘Actual Component’) is set at the relative proportion of the point yielding the maximum amount of cellobiose, as predicted by the statistical model.

**(A)**

**
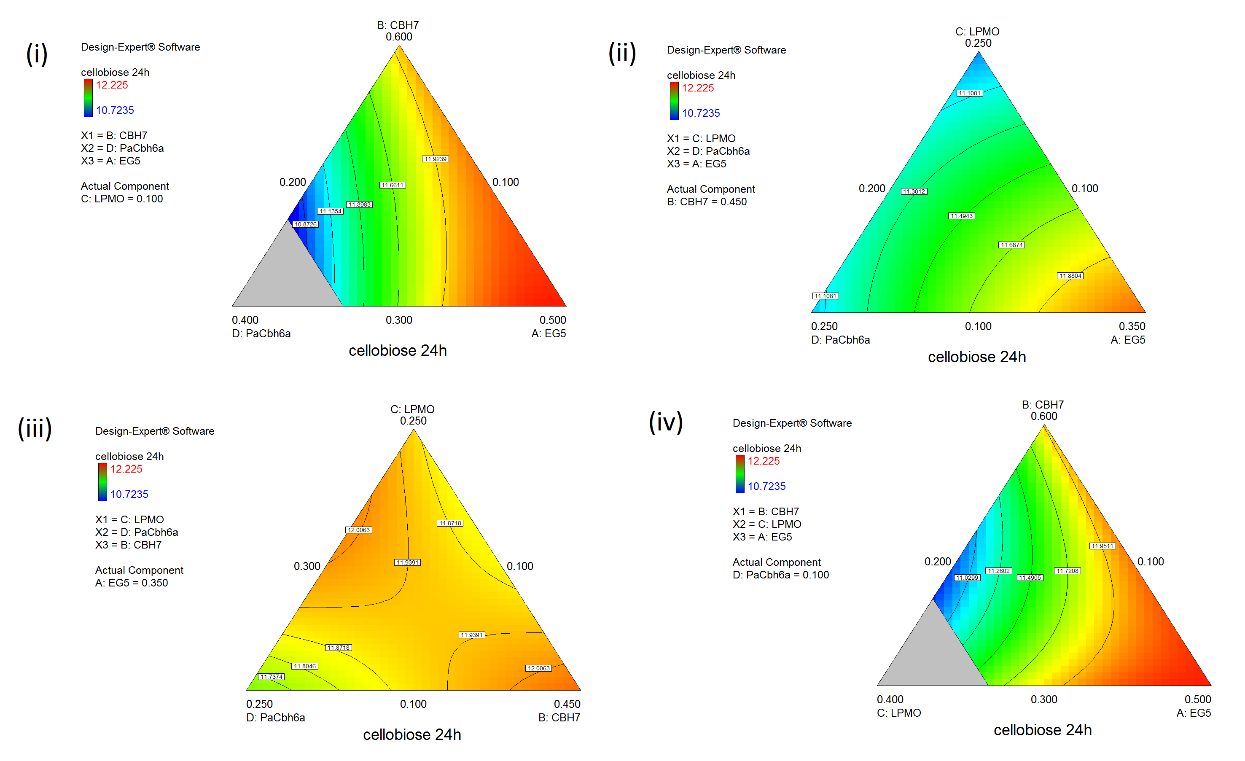
**

**(B)**

**
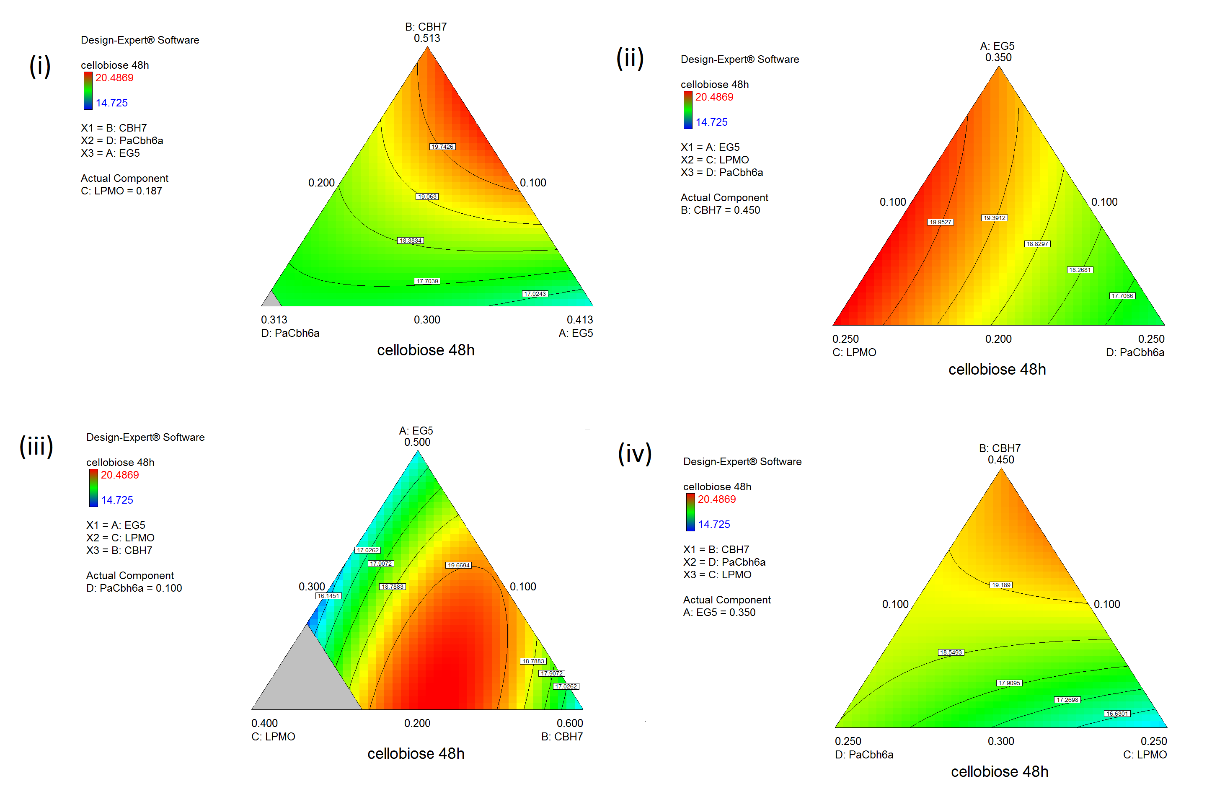
**
